# Unraveling multi-state molecular dynamics in single-molecule FRET experiments. II. Quantitative analysis of multi-state kinetic networks

**DOI:** 10.1063/5.0095754

**Published:** 2022-07-21

**Authors:** Oleg Opanasyuk, Anders Barth, Thomas-Otavio Peulen, Suren Felekyan, Stanislav Kalinin, Hugo Sanabria, Claus A. M. Seidel

**Affiliations:** 1 Institut für Physikalische Chemie, Lehrstuhl für Molekulare Physikalische Chemie, Heinrich Heine Universität, Düsseldorf, Germany; 2 Department of Physics and Astronomy, Clemson University, Clemson, South Carolina 29634, USA

## Abstract

Single-molecule Förster Resonance Energy Transfer (smFRET) experiments are ideally suited to resolve the structural dynamics of biomolecules. A significant challenge to date is capturing and quantifying the exchange between multiple conformational states, mainly when these dynamics occur on the sub-millisecond timescale. Many methods for quantitative analysis are challenged if more than two states are involved, and the appropriate choice of the number of states in the kinetic network is difficult. An additional complication arises if dynamically active molecules coexist with pseudo-static molecules in similar conformational states with undistinguishable Förster Resonance Energy Transfer (FRET) efficiencies. To address these problems, we developed a quantitative integrative analysis framework that combines the information from FRET-lines that relate average fluorescence lifetimes and intensities in two-dimensional burst frequency histograms, fluorescence decays obtained by time-correlated single-photon-counting, photon distribution analysis of the intensities, and fluorescence correlation spectroscopy. Individually, these methodologies provide ambiguous results for the characterization of dynamics in complex kinetic networks. However, the global analysis approach enables accurate determination of the number of states, their kinetic connectivity, the transition rate constants, and species fractions. To challenge the potential of smFRET experiments for studying multi-state kinetic networks, we apply our integrative framework using a set of synthetic data for three-state systems with different kinetic connectivity and exchange rates. Our methodology paves the way toward an integrated analysis of multiparameter smFRET experiments that spans all dimensions of the experimental data. Finally, we propose a workflow for the analysis and show examples that demonstrate the usefulness of this toolkit for dynamic structural biology.

## I. ANALYSIS OF MULTI-STATE KINETIC NETWORKS

Biomolecular dynamics are often complex, involving multiple conformational states and sub-states that interconvert over a wide range of timescales from nanoseconds to minutes and hours. Single-molecule FRET (smFRET) experiments provide a wealth of information about the molecular system and are ideal for resolving these dynamics.^1–4^ Various analysis methods have been developed over the years to obtain quantitative information on the structural dynamics of the biomolecular systems in smFRET experiments of surface-immobilized or freely diffusing molecules (reviewed in Ref. 1).

In this work, we focus on smFRET experiments performed on freely diffusing molecules using multiparameter fluorescence detection (MFD), where the structural and dynamic information is encoded in the time-ordered sequence of the detected photons recorded with picosecond resolution.^5^ The most widely used methods for this measurement modality are the statistical analysis of Förster Resonance Energy Transfer (FRET)-efficiency histograms (photon distribution analysis, PDA),^6–10^ intensity-based fluorescence correlation spectroscopy (FCS),^11–17^ and time-resolved fluorescence decay analysis [time-correlated single photon counting (TCSPC)],^18–20^ but other approaches have been applied as well.^21–23^ Each representation of the experimental data and the corresponding analysis method has its strengths and weaknesses to determine the fluorescence properties of the species, detect their kinetic connectivity, and quantify the rate constants (Table I). Established methods such as FCS and TCSPC are computationally fast. They rely on established algorithms to find the optimal parameters of a physical or empirical model that describe the experimental data. While TCSPC is ideally suited to resolve the FRET efficiencies of the contributing states, conformational dynamics from nano- to milliseconds can be resolved by fluorescence correlation spectroscopy (FCS) and its extensions fluorescence lifetime correlation spectroscopy (FLCS),^24^ two-dimensional FLCS,^25^ and filtered-FCS (fFCS), which utilize statistical weighting to recover species-specific correlation curves.^13,26^ These correlation approaches work well for homogeneous samples of dynamic molecules interconverting between two conformational states. However, they are challenged by the increased complexity of many biological systems that involve three or more states interconverting on different kinetic timescales or contain heterogeneous mixtures of static and dynamic molecules. In such complex situations, statistical analysis of the shape and width of peaks in FRET efficiency histograms by dynamic PDA^8^ can provide important complementary information. Therefore, to unravel the complex dynamics of such systems, a holistic approach combining multiple methods is required.

**TABLE I.** Comparison of methods to analyze conformational dynamics of multi-state kinetic networks in smFRET experiments. The methods are compared to their ability to identify the different states (fluorescence properties and fractions), kinetic connectivity, dynamic exchange, and the accessible timescales. For cases where no detailed text is given, a “+” indicates that the method is well suited for a particular task, a “⋯” indicates that a method is insensitive to a certain parameter, and a “o” means that the given information can be obtained in principle if careful controls are performed. MLE: Gopich-Szabo photon trajectory analysis using maximum likelihood estimation,^35^ BVA: burst variance analysis,^36^ FRET-2CDE: FRET two-channel kernel density estimator.^37^

Method	Identification of states	Kinetic connectivity	Quantification of dynamics	Accessible timescale
TCSPC	+	⋯	⋯	⋯
FCS	⋯	⋯	+	*μ*s to ms
MLE	o	o	+	*μ*s to ms^a^
PDA/histogram analysis	For slow dynamics^b^	⋯	+	100 *µ*s to 10 ms^a^
BVA/FRET-2CDE	For slow dynamics^b^	+	Qualitatively	100 *µ*s to 10 ms^a^
2D histogram: *E* vs ${\left\langle \tau_{D\left(A\right)}\right\rangle }_{F}$	For slow dynamics^b^	+	Qualitatively	*µ*s to ms

^a^ The lower limit depends on the average inter-photon time. Faster timescales are accessible for higher signal count rates.

^b^ Timescale of dynamics ≥500 *μ*s.

Existing methods for the quantitative analysis of dynamics are applied to a reduced representation of the single-photon-counting data. At the same time, the full potential of the multidimensional dataset is not utilized. This multidimensional information is revealed in the pairwise histograms of averaged fluorescence observables—although the informational content is likewise reduced due to the averaging performed over each single-molecule event.^27^ One example of how the multidimensional information can be utilized is the pairwise plot of the intensity-based FRET efficiency *E* and the intensity-weighted average donor fluorescence lifetime ${\left\langle \tau_{D\left(A\right)}\right\rangle }_{F}$. The correlation between these two FRET indicators enables the detection of conformational dynamics by revealing the exchange between different states, providing graphical information on the connectivity within the kinetic network (as described in Paper I of this Tutorial series by Barth *et al.*^28^). The concept of these FRET-lines was first introduced by Rothwell *et al.*^29^ and Margittai *et al.*^30^ and expanded to dynamic exchange by Kalinin *et al.*^8^ Barth *et al.*^28^ presented a generalized theory of the FRET-lines, provided various software tools to generate FRET-lines, and discussed a large variety of use cases for dynamics exchange between multiple ordered and disordered conformational states.

Ideally, a multi-state kinetic model would be directly fit to this multidimensional dataset. However, to our knowledge, a quantitative description of the complete multidimensional histogram is currently limited to computationally expensive stochastic simulations. The stochastic nature of Monte Carlo simulations also makes this approach difficult to apply in optimization routines, which converge more rapidly if analytical expressions are employed. While such expressions are known for simple cases,^31^ they are currently unavailable for the multi-state networks discussed in this work.

Here, we take a step toward a holistic analysis framework by using FRET-lines as pathfinders and by combining them with TCSPC, distinct FCS techniques, and PDA in a global approach to quantify the exchange in multi-state kinetic networks. In a first step, the correct kinetic model is identified by a graphical analysis using FRET-lines, defining the number of FRET species and their linkage. The exchange rates are then quantified using a global analysis of the donor fluorescence decay and the color correlation functions. The framework is applied to simulated datasets of multi-state systems with a binary exchange between two species in the presence of a background of static molecules. When only the TCSPC and FCS information is used, ambiguous solutions are obtained that differ in the kinetic connectivity of the species and the fraction of molecules participating in the dynamic exchange. To resolve this ambiguity, FRET-lines provide a graphical analysis of the kinetic connectivity of species and permit the estimation of the equilibrium constant from the peak of the dynamic population in binary systems. For systems involving a fast dynamic exchange between more than two species, additional information is required. Using simulations of three-state systems, we illustrate the potential of filtered-FCS to detect the direct exchange between different species in complex networks and deduce the kinetic linkage, even in this challenging case. Finally, we derive relations between the correlation amplitudes and the single-molecule FRET indicators *E* and ${\left\langle \tau_{D\left(A\right)}\right\rangle }_{F}$, highlighting the connections between the different representations of the data and the future possibility to extend this holistic approach to data analysis.

## II. NOMENCLATURE FOR MULTI-STATE SYSTEMS IN SMFRET EXPERIMENTS

**FIG. 1.**
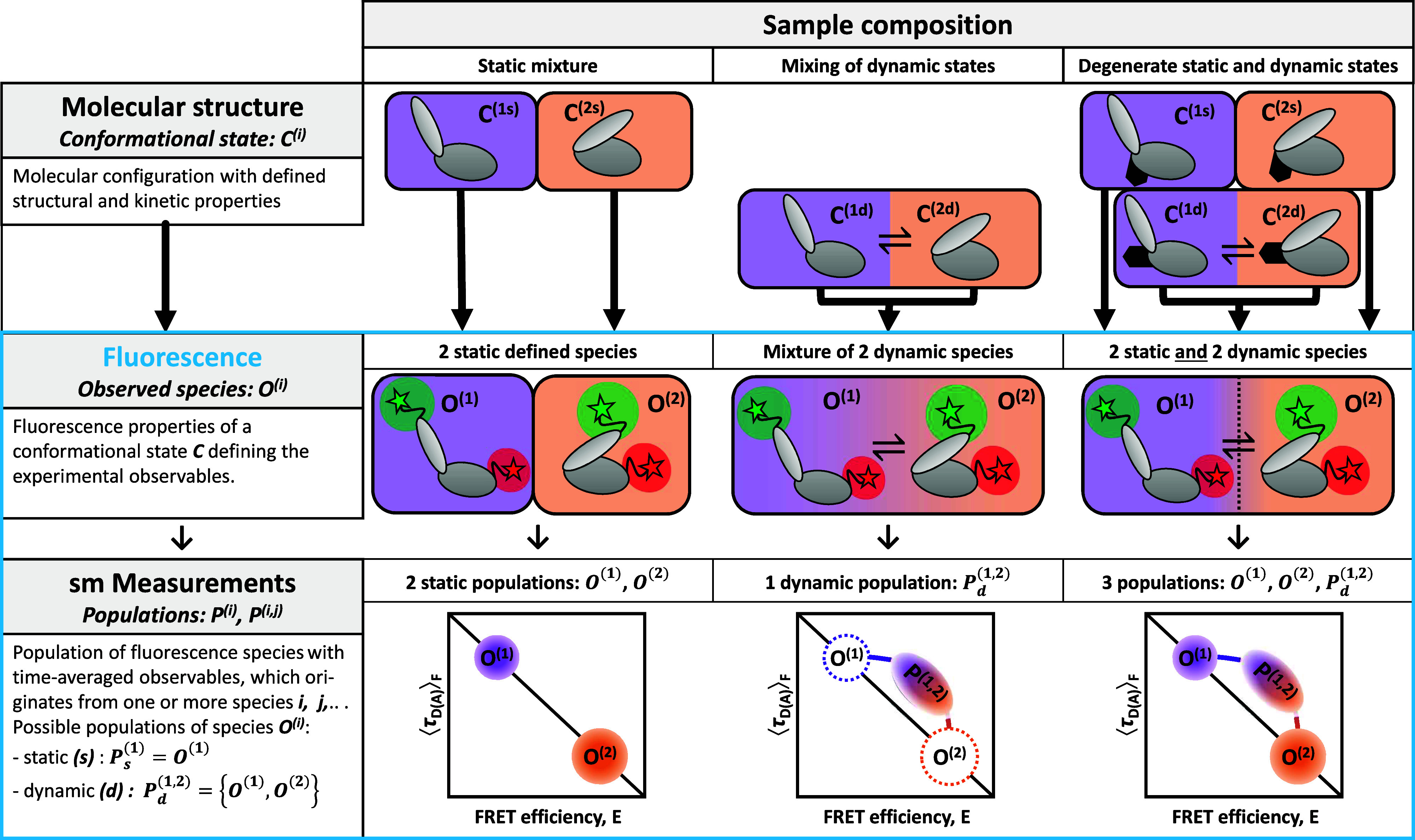
From structural states to fluorescence populations. Definitions in smFRET experiments of multi-state systems. Top row: A conformational state is defined as a distinct structural state of a biomolecule, which may be found in dynamic exchange with other states or trapped in a static configuration. The dynamic behavior may be controlled by a conformational switch in an associated domain that is not probed by FRET (black). Middle row: A fluorescence species is defined by the fluorescence properties, such as the FRET efficiency, fluorescence lifetime, and fluorescence anisotropy, of the donor and acceptor dyes. All members of a fluorescent species have identical fluorescence properties. Different conformational states may belong to the same fluorescent species if the structural change does not affect the properties of the fluorescent probe. Different fluorescent species are often found to belong to a single conformational state, e.g., due to interactions of the fluorophore with the biomolecular surface. Transient quenching or binding of the dyes to the surface would, for example, result in different fluorescent species that relate to the same conformational state (see the supplementary material, Note 1, for details). The assignment of fluorescent species to conformational states is an interpretative step in the analysis and usually requires prior structural knowledge. Bottom row: On the level of the experiment, one observes populations in the one- or two-dimensional histograms of the FRET efficiency *E* against the donor fluorescence lifetime ${\left\langle \tau_{D\left(A\right)}\right\rangle }_{F}$. Populations are clusters of single-molecule events with identical observed fluorescence properties. A population may represent a single fluorescence species or, in the case of fast exchange between different fluorescent species, may originate from a heterogeneous mixture of different fluorescence species. Populations that fall on the static FRET-line (black diagonal line) represent a single fluorescence species, while shifted populations indicate dynamic exchange. Heterogeneity within the population may be revealed by a sub-ensemble analysis, e.g., of the fluorescence decay. The populations surrounded by dashed lines in the middle panel indicate the hypothetical position of the static populations of the two conformational states *O*^(1)^ and *O*^(2)^ that mix within the dynamic population *P*^(1,2)^ for the fast conformational exchange.

Purely static or dynamic biomolecules are rarely found in nature. It is often observed that biomolecules can be activated through allosteric effects, such as binding of cofactors or regulators, posttranslational modifications, or conformational changes in associated domains, switching the molecule from a static into a dynamic state.^4,32–34^ In such situations, molecules with the same FRET efficiency may either be static or participate in the conformational dynamics, introducing a degeneracy into the analysis in which the same observed FRET species may belong to different states that are either static or dynamic. Moreover, the conformational space of biomolecules is huge, so that their dynamic behavior is often modulated by conformational switches in associated domains (Fig. 1, top row: small black domain) that are not probed by FRET. This has been observed, for example, in nucleosomes^32^ and chromatin arrays.^4^ Note that while nothing in biology is truly static, here, we refer to *pseudo-static* populations of molecules with structural dynamics on timescales that are much longer (>100 ms) than the typical observation time in single-molecule experiments of freely diffusing molecules of ∼1 ms.

In FRET experiments on freely diffusing single molecules, the accessible timescales of dynamics are limited by the diffusion time to <10 ms, causing additional complications because transitions between conformational states on slower timescales could appear as *pseudo-static* populations in the analysis. To avoid confusion about the physical description of the biomolecular system as static or dynamic and to classify observed populations in the experiment, we propose a concise nomenclature for smFRET experiments in Fig. 1. A *conformational state C*^(*i*)^ is defined as a distinct structural state of the biomolecule that can be classified as static or be in dynamic exchange with other conformational states. The alternation between dynamic and static states of the biomolecule may be subject to allosteric regulation, biomolecular interactions, or covalent modifications. In the smFRET experiment, conformational states are observed indirectly through the fluorescence properties of the covalently linked dyes, such as the FRET efficiency, fluorescence lifetime, or fluorescence anisotropy of the donor and acceptor fluorophores. An *observed fluorescence species O*^(*i*)^ is generally assigned to a single conformational state. However, due to quenching or sticking of the fluorophores, different fluorescence species may belong to the same conformational state. On the other hand, multiple conformational states may belong to the same fluorescence species if the fluorescence properties do not change significantly (see the supplementary material, Note 1, for an overview of potential ambiguities). In smFRET experiments, fluorescence species are observed as *populations P*^(*i*)^ in the one- or two-dimensional histograms. Due to dynamic averaging during the diffusion time, a population may originate from a mixture of different fluorescence species. Dynamic and static populations may be distinguished in a plot of the FRET efficiency *E* against the donor fluorescence lifetime ${\left\langle \tau_{D\left(A\right)}\right\rangle }_{F}$ (Fig. 1, bottom). The static populations, originating from the fluorescence species *O*^(1)^ and *O*^(2)^, lie on the static FRET-line. In contrast, the dynamic population *P*^(1,2)^ shows the characteristic dynamic shift (ds) from the static FRET-line, as introduced in Paper I.^28^ The heterogeneity within the dynamic populations can be resolved by a sub-ensemble analysis of the fluorescence decays.

The assignment of static and dynamic populations is complicated when the sample contains a mixture of static and dynamic conformational states of identical FRET efficiencies (Fig. 1, right). In the limit of fast dynamic exchange, a dynamic population is shifted from the static FRET-line and separated from static populations. As the conformational exchange becomes slower and approaches the diffusion time of the molecule, there is a probability that dynamic molecules do not undergo a conformational change during the observation time. While originating from dynamic conformational states, these single-molecule events will show as a *pseudo-static* population on the static FRET-line and are difficult to separate from actual static populations. As shown below, the fraction of dynamic molecules is a central parameter in the quantitative analysis of such heterogeneous multi-state systems by correlation methods. In Sec. III, we will first show how a graphical analysis can be employed to estimate the equilibrium constant of the dynamic exchange in the background of static species.

## III. GRAPHICAL ANALYSIS OF DYNAMIC POPULATIONS

Quantitative information on conformational dynamics is encoded in the shape of the FRET efficiency histogram, as in dynamic photon distribution analysis (PDA).^8,10,38^ These analyses, however, are challenged if the experiment contains a mixture of static and dynamic molecules due to the difficulty of distinguishing actual static and pseudo-static molecules. Pseudo-static molecules are dynamic molecules that, by chance, remained in one conformational state during the transit through the observation volume. This section will describe how the separation of static and dynamic molecules in the *E*–${\left\langle \tau_{D\left(A\right)}\right\rangle }_{F}$ histogram can provide quantitative information on the equilibrium constant by a graphical analysis of the peak of the dynamic population.

In the description of FRET-lines, the timescales of the dynamics are not considered explicitly. For a dynamic system, the distribution of the state occupancies *x*^(*i*)^ depends on the microscopic exchange rates and the observation time.^8,31,39^ For the calculation of dynamic FRET-lines in Paper I,^28^ we have instead considered all possible values for the state occupancy, *x*^(1)^ ∈ {0, 1}. In other words, we have replaced the true distribution of the state occupancies by a uniform distribution with equal probability for all values of *x*^(1)^ [$p\left(x^{\left(1\right)}\right)=const$]. FRET-lines may, however, still be used to address the timescale of dynamics qualitatively. In the absence of dynamics, the two-dimensional histogram will reveal distinct static populations as limiting species, which fall onto the static FRET-line. In the case of fast exchange between distinct FRET species, the conformational dynamics are averaged for every single-molecule event, resulting in a single population representing the equilibrium. “Fast” exchange relates to the timescale of diffusion (∼1–5 ms) and generally classifies processes on a timescale of 100 *µ*s and below. This single peak will be shifted from the static FRET-line in the two-dimensional histogram, as described before. The slow transition between limiting species, such as the dynamics on the timescale of diffusion or slower, leads to a broadening of the observed distributions; thus, the shape of the distribution depends on the timescale of the dynamics.

**FIG. 2.**
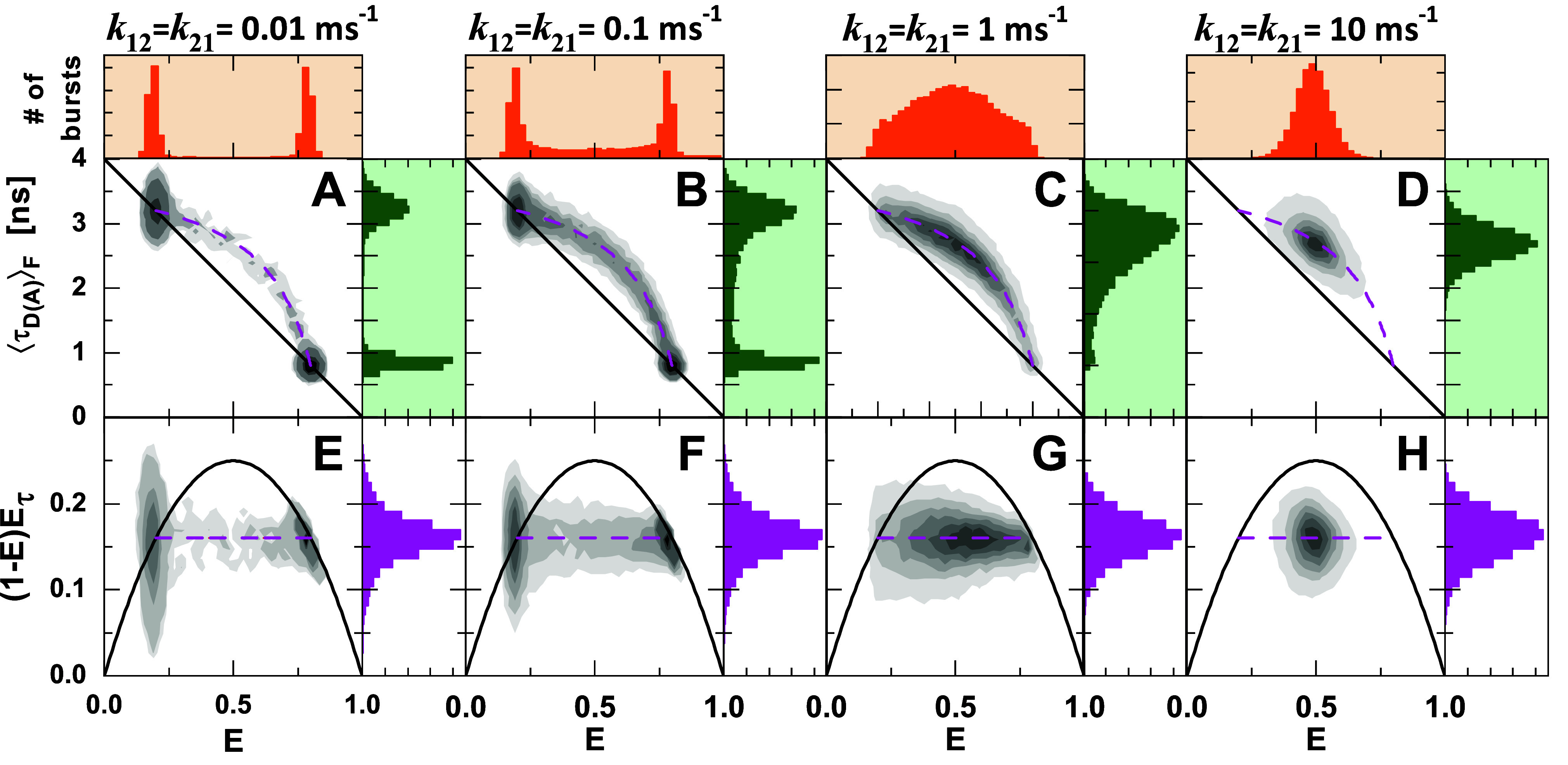
Simulated smFRET experiments for a two-state system exchanging at increasing exchange rates between states with FRET efficiencies of E_1_ = 0.2 and E_2_ = 0.8. Shown are the two-dimensional histograms of ${\left\langle \tau_{D\left(A\right)}\right\rangle }_{F}$ vs E (a)–(d) and the difference between the first and second moments of the lifetime distribution, $\left(1-E\right)E_{\tau }$vs E (e)–(h) for different timescales of conformational dynamics from slow (*k*_12_ = *k*_21_ = 0.01 ms^−1^) to fast exchange (*k*_12_ = *k*_21_ = 10 ms^−1^). The population's shape and location indicate the dynamic timescale, while the dynamic FRET-line is independent of the rate constants and describes all timescales. The diffusion time in all simulations is t _diff_ = 1.5 ms.

To illustrate this effect, we performed simulations of a two-state system with FRET efficiencies of 0.2 and 0.8 (Fig. 2). We set the backward and forward rates equal (*k*_12_ = *k*_21_) and varied them from 0.01 to 10 ms^−1^, at a constant diffusion time *t*_diff_ = 1.5 ms. When the rate constants are significantly slower than the inverse diffusion time, 1/*t*_diff_ = 0.67 ms^−1^, the two subpopulations are separated because molecules do rarely interconvert during the observation time [Figs. 2(a) and 2(e)]. With increasing rate constants, the molecules are more likely to change their state during the observation time, resulting in single-molecule events with intermediate FRET efficiencies [Figs. 2(b), 2(c), 2(f), and 2(g)], while complete averaging is observed at fast exchange rates [Figs. 2(d) and 2(h)]. However, the dynamic FRET-line describes all possible mixing ratios between the involved species, regardless of the magnitude of the rate constants, and applies to all cases.

**FIG. 3.**
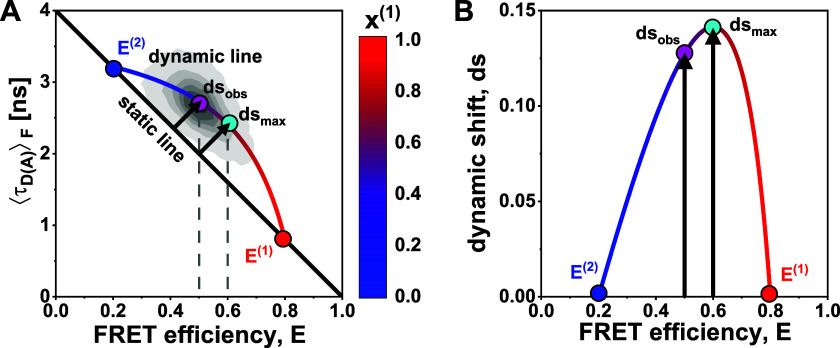
The dependence of the observed dynamic shift on the average FRET efficiency. (a) The dynamic FRET-line for a two-state system with FRET efficiencies E ^(1)^ = 0.8 (red) and E ^(2)^ = 0.2 (blue) reaches a maximum dynamic shift, ds_max_ (cyan), as indicated by the arrow, at a FRET efficiency of E = 0.6, corresponding to a species fraction of x ^(1)^ = 2/3. A smaller dynamic shift, ds_obs_ (magenta), is observed for the simulation with rate constants of *k*_12_ = *k*_21_ = 10 ms^−1^ [x ^(1)^ = 0.5, compare Fig. 2(d)]. The static FRET-line is given in black, and the dynamic FRET-line is color-coded according to the species fraction x ^(1)^. (b) The dependence of the dynamic shift on the average FRET efficiency E for the two-state system shown in panel (a).

**FIG. 4.**
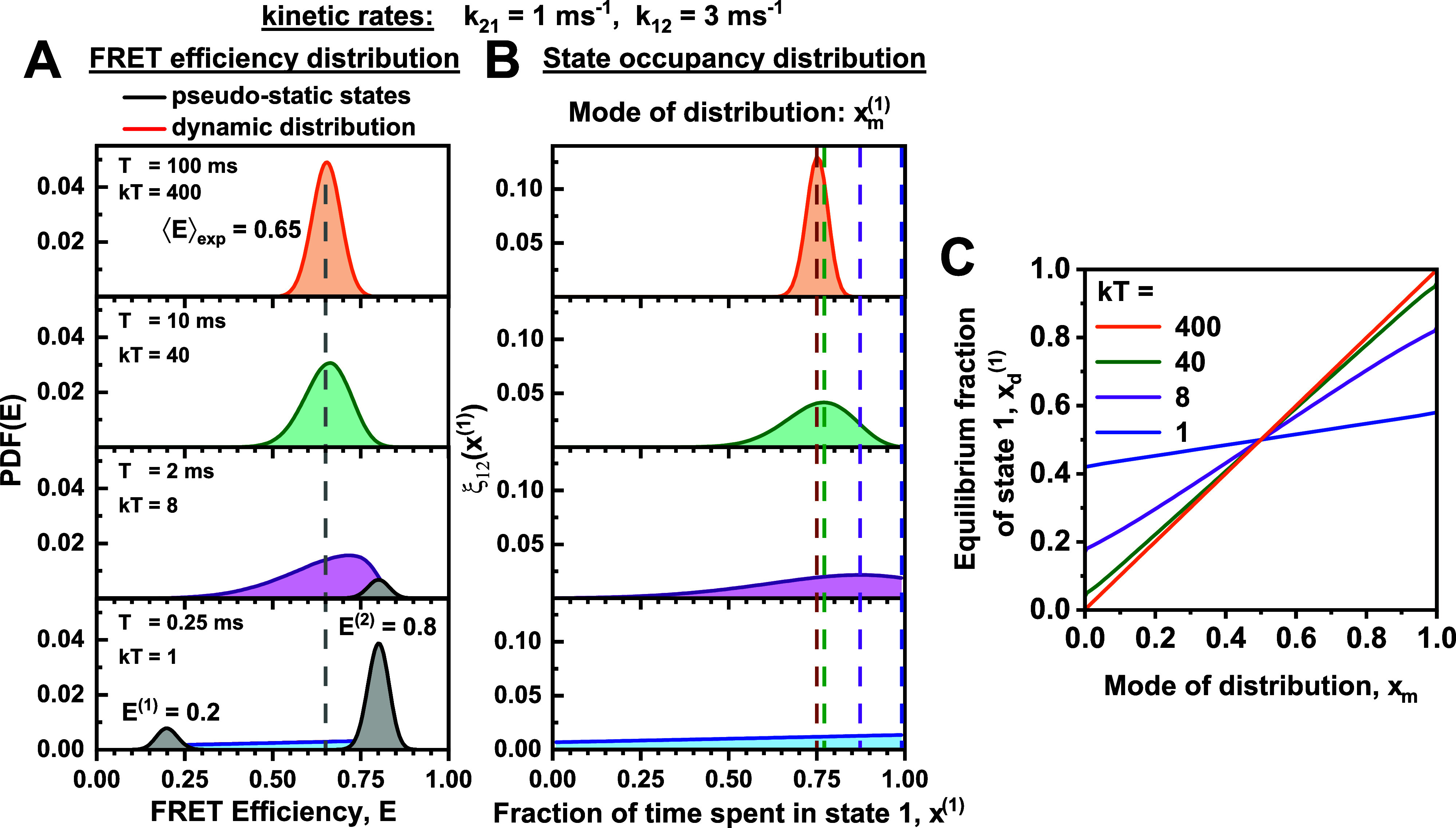
Extracting equilibrium fractions from the dynamic population for a two-state system. (a) Simulated FRET efficiency distributions for a two-state system with exchange rates of k_21_ = 1 ms^−1^ and k_12_ = 3 ms^−1^ and FRET states of E^(1)^ = 0.2 and E^(2)^ = 0.8 at varying observation times T. The distribution is split into the pseudo-static contribution of molecules that did not interconvert during the observation time (black) and the dynamic contribution of molecules that showed one or more transitions (red). The average FRET efficiency ⟨E⟩_exp_ = 0.65 is independent of the observation time and reflects the equilibrium between the two species. (b) The dynamic part of the distribution of the occupancy of state 1, $\xi \left(x^{\left(1\right)}\right)$, of the FRET efficiency histograms shown in (a). At shorter observation times, the mode of the distribution x_m_ deviates from the equilibrium fraction $x_{d}^{\left(1\right)}=\frac{k_{12}}{k_{12}+k_{21}}=0.75$. The colored dashed vertical lines indicate the modal values $x_{m}^{\left(1\right)}$ for the state occupancy distributions corresponding to the given integration times (orange: 100 ms, green: 10 ms, purple: 2 ms, blue: 0.25 ms). (c) The relationship between the mode of the exchange distribution and the equilibrium fraction is found to be approximately linear. This allows for a direct conversion of the measured modal value into the equilibrium fraction if the sum of the rates, k = k_12_ + k_21_, is known.

In Paper I ofthis Tutorial series,^28^ we defined the observed deviation of a population perpendicular to the static FRET-line as the dynamic shift [Fig. 3(a)]. Interestingly, the dynamic shift of the population for the simulated system with *k*_12_ = *k*_21_ does not reach its maximum possible value even for fast dynamics [*k*_12_ = *k*_21_ = 10 ms^−1^, Figs. 2(d) and 3(a)]. In the limiting case of fast dynamics, the observed dynamic shift of the dynamically averaged population depends on the FRET efficiencies ($E^{\left(1\right)}$ and $E^{\left(2\right)}$) and the species fractions (*x*^(1)^ and *x*^(2)^) of the two states (see the supplementary material, Note 2):

$$ds\left(x^{\left(1\right)}\right)\;=\;\frac{1}{\sqrt{2}}{\left(E^{\left(2\right)}\;-\;E^{\left(1\right)}\right)}^{2}\frac{x^{\left(1\right)}\left(1-x^{\left(1\right)}\right)}{x^{\left(1\right)}\left(1-E^{\left(1\right)}\right)+\left(1-x^{\left(1\right)}\right)\left(1-E^{\left(2\right)}\right)},$$ (1)

where *x*^(1)^ is the species fractions of state 1. The dependence of the observed dynamic shift on the average FRET efficiency of the dynamic population, *E*, is illustrated in Fig. 3(b). The maximum dynamic shift, ds_max_, is given by (see Paper I, Fig. 4^28^)

$$ds_{\max}=\frac{1}{\sqrt{2}}{\left(\sqrt{1-E^{\left(1\right)}}-\sqrt{1-E^{\left(2\right)}}\right)}^{2},$$ (2)

which is obtained at a species fraction $x_{\max}^{\left(1\right)}$ of

$$x_{\max}^{\left(1\right)}=\frac{\sqrt{1-E^{\left(2\right)}}}{\sqrt{1-E^{\left(1\right)}}+\sqrt{1-E^{\left(2\right)}}},$$ (3)

corresponding to a value of $x_{\max}^{\left(1\right)}=2/3$ in the given example. The maximum dynamic shift defined by Eq. (2) thus serves as a figure of merit to judge whether dynamics could be detected in the experiment in the ideal case, while the observed value of the dynamic shift also depends on the equilibrium constant according to Eq. (1).

Beyond the qualitative assessment of dynamics by visual inspection of the two-dimensional histogram, it is possible to extract quantitative information about the dynamic equilibrium from the two-dimensional histograms. The equilibrium species fractions $x_{d}^{\left(1\right)}$ and $x_{d}^{\left(2\right)}$ (i.e., the fraction of molecules in state 1 or 2) can be determined from the average FRET efficiency for all single-molecule events, ⟨*E*⟩_exp_, by

$$x_{d}^{\left(1\right)}=\frac{k_{12}}{k_{21}+k_{12}}=\frac{{\left\langle E\right\rangle }_{\exp}-E^{\left(2\right)}}{E^{\left(1\right)}-E^{\left(2\right)}};\;\;x_{d}^{\left(2\right)}=1-x_{d}^{\left(1\right)},$$ (4)

where $E^{\left(1\right)}$ and $E^{\left(2\right)}$ are the FRET efficiencies of the limiting species. From the species fractions, the equilibrium constant can be calculated, which relates to the kinetic rates by

$$K=\frac{x_{d}^{\left(2\right)}}{x_{d}^{\left(1\right)}}=\frac{k_{21}}{k_{12}}.$$ (5)

While for purely dynamic systems ⟨*E*⟩_exp_ is readily calculated from the experimental dataset, the presence of additional static species will result in incorrect values for the species fractions. In the case of a mixture of dynamic and static molecules, it would be advantageous if the equilibrium fraction could be obtained from the position of the dynamic population alone, which is most easily defined by its peak value or mode. For fast exchange, the mode of the population in the two-dimensional histogram directly corresponds to the average FRET efficiency ⟨*E*⟩_exp_. If the timescale of kinetics becomes comparable to the diffusion time, the mode of the dynamic distribution deviates from the actual value of the species fraction *x*^(1)^. To study this effect, we consider the distribution of FRET efficiencies for a dynamic system explicitly. The average FRET efficiency within a single-molecule event, *E*, depends on the fraction of time spent in the different states, equivalent to the species fractions *x*^(*i*)^:

$$E=x^{\left(1\right)}E^{\left(1\right)}+\left(1-x^{\left(1\right)}\right)E^{\left(2\right)}.$$ (6)

Here, *x*^(1)^ is the species fraction of state 1 in a single-molecule event, which is different from the equilibrium species fraction $x_{d}^{\left(1\right)}$ discussed before. If we know the state occupancy distribution of *x*^(1)^, $P\left(x^{\left(1\right)}\right)$, we can calculate the distribution of FRET efficiencies *P*(*E*). In general, $P\left(x^{\left(1\right)}\right)$ takes a complex mathematical form (a complete derivation is given in the supplementary material, Note 3), but it can be simplified to the sum of three terms:

$$\begin{array}{ll} P\left(x^{\left(1\right)}\right) & =\frac{k_{12}}{k_{12}+k_{21}}e^{-k_{21}T}\delta \left(1-x^{\left(1\right)}\right)\\ & \;+\;\frac{k_{21}}{k_{12}+k_{21}}e^{-k_{12}T}\delta \left(x^{\left(1\right)}\right)+\xi_{12}\left(x^{\left(1\right)}\right). \end{array}$$ (7)

Here, the first and the second terms describe the probability that the molecule was in state 1 or 2 at the beginning of the observation time *T* and did not interconvert (pseudo-static states). Moreover, the term $\xi_{12}\left(x^{\left(1\right)}\right)$ describes the dynamic part of the distribution of *x*^(1)^, such as in the case that molecules switched between the states at least once during its observation time. This term is given by^8,39–41^

$$\begin{array}{ll} \xi_{12}\left(x^{\left(1\right)}\right) & =\frac{T}{\frac{1}{k_{12}}+\frac{1}{k_{21}}}\times e^{-\left(k_{12}x^{\left(2\right)}+k_{21}x^{\left(1\right)}\right)T}\\ & \;\times \;\left[2I_{0}\left(2T\sqrt{k_{21}k_{12}x^{\left(1\right)}x^{\left(2\right)}}\right)\right.\\ & \;\left.+\;\frac{x^{\left(1\right)}k_{12}+x^{\left(2\right)}k_{21}}{\sqrt{k_{21}k_{12}x^{\left(1\right)}x^{\left(2\right)}}}I_{1}\left(2T\sqrt{k_{21}k_{12}x^{\left(1\right)}x^{\left(2\right)}}\right)\right], \end{array}$$ (8)

where $x^{\left(2\right)}=1-x^{\left(1\right)}$ and *I*_0_ and *I*_1_ are the modified Bessel functions of the first kind of order zero and one, respectively. For fast exchange (or long observation times), the pseudo-static terms vanish while only the dynamic term remains. On the other hand, for slow dynamics, the static terms tend to the equilibrium fractions of the two states, while the dynamic term vanishes.

Theoretical FRET efficiency distributions for a two-state dynamic system are given in Fig. 4(a) for rate constants o *k*_21_ = 1 ms^−1^ and *k*_12_ = 3 ms^−1^ at different observation times *T*. For short observation times (*T* = 0.25 ms), only the pseudo-static peaks remain, while complete averaging is observed for long observation times (*T* = 100 ms). The average FRET efficiency ⟨*E*⟩_exp_ of 0.65 relates to the equilibrium species fractions by Eq. (4). Considering the case that additional static species contribute to the average FRET efficiency, we would like to infer the equilibrium species fraction only from the dynamic part of the distribution $\xi_{12}\left(x^{\left(1\right)}\right)$ [Fig. 4(b)]. The property of $\xi_{12}\left(x^{\left(1\right)}\right)$ that is most easily inferred from the two-dimensional histograms is its maximum or peak (compare Fig. 2). For an observation time *T* = 100 ms, the modal value $x_{\text{m}}^{\left(1\right)}$ corresponds to the equilibrium fraction of state 1, *x*^(1)^ = 0.75 [Fig. 4(b), top]. However, as the observation time decreases, $x_{\text{m}}^{\left(1\right)}$ deviates from the equilibrium fraction to the point where the modal value coincides with the pseudo-static population [$x_{\text{m}}^{\left(1\right)}$ = 1, Fig. 4(b) bottom]. Fortunately, the relationship between the actual equilibrium fraction of state 1, $x_{d}^{\left(1\right)}$, and the modal value of the distribution, $x_{\text{m}}^{\left(1\right)}$, is approximately linear, enabling a simple conversion between the two quantities by [Fig. 4(c) and the supplementary material, Note 3]

$$x_{d}^{\left(1\right)}\approx x_{d,\lim}^{\left(1\right)}+\left(1-2x_{d,\lim}^{\left(1\right)}\right)x_{m}^{\left(1\right)},$$ (9)

where $x_{d,\lim}^{\left(1\right)}$ is the limiting equilibrium fraction at *x*_*m*_ = 0 [i.e., the ordinate intercept in Fig. 4(c)] that depends only on the average number of transitions during the observation time given by $\left(k_{21}+k_{12}\right)T$:

$$x_{d,\lim}^{\left(1\right)}\left(kT\right)=\frac{3}{2}{\left(1+\frac{kT}{2}\left(1+\frac{I_{0}\left(\frac{kT}{2}\right)}{I_{1}\left(\frac{kT}{2}\right)}\right)\right)}^{-1},$$ (10)

where *k* = *k*_21_ + *k*_12_.

The modal value of the species fraction, $x_{\text{m}}^{\left(1\right)}$, can be obtained from the modal value of the FRET efficiency distribution, *E*_*m*_, obtained by graphical analysis if the FRET efficiencies of the limiting states are known:

$$x_{m}^{\left(1\right)}=\frac{E_{m}-E^{\left(2\right)}}{E^{\left(1\right)}-E^{\left(2\right)}}.$$ (11)

If the sum of rates *k* is known from FCS analysis or other methods, the equilibrium fraction and the equilibrium constant can be determined from Eqs. (9) and (10), which allows determining the microscopic rate constants quantitatively using the equilibrium information obtained from the graphical analysis of the two-dimensional histograms. While the approach in principle requires observation time windows of identical length *T*, it may, as an approximation, be set to the diffusion time for datasets of single-molecule events of freely diffusing molecules.

## IV. GLOBAL ANALYSIS OF MULTI-STATE DYNAMICS

### A. Analytical description of FCS curves

Fluorescence correlation spectroscopy (FCS) relies on the fluctuations of recorded signals to characterize molecular interactions, such as binding and unbinding, chemical kinetics, and diffusion of fluorescent molecules.^11,12,42^ Importantly, when combined with FRET, FCS enables a quantitative analysis of conformational dynamics.^15,43–46^ Typically, the fluorescence signals are collected over specific spectral detection windows. Here, we refer to the correlation analysis of the fluorescence intensities of a donor and an acceptor fluorophore (monitored in two detection channels commonly named “green” and “red”) as color-FCS. We avoid the conventional abbreviation FRET-FCS to differentiate it from the related method of filtered-FCS (fFCS), which relies on FRET to distinguish different species but does not explicitly use color channels. Analytical models for color-FCS are usually limited to kinetic networks involving two states due to the increased number of parameters of multi-state systems and the limited experimental information available.^45^ Advanced correlation methods take advantage of the lifetime information available with pulsed laser excitation, allowing one to interrogate biomolecular dynamics by two-dimensional maps of fluorescence decays^47,48^ or filtered correlation algorithms.^13,26,49,50^ By using an additional dimension of the collected data, these methods offer the potential to interrogate more complex kinetic networks. Quantitative analysis of FCS experiments requires a set of model-specific analytic functions that describe the time evolution of the correlations. Often, smFRET and FCS experiments are used to study unimolecular reactions, wherein a biomolecule switches between different conformational states during the observation time. These dynamic molecules may be found together with molecules that are stable on the timescale of seconds to minutes and are, thus, considered as static in the single-molecule experiment.

In our model, we, thus, consider the coexistence of static and dynamic molecules with identical properties of the respective conformational states (Fig. 1). Dynamic molecules may change between different conformational states during the observation time, resulting in variations of the fluorescence properties, such as the FRET efficiency and donor fluorescence lifetime, while these properties remain constant for static molecules. For unimolecular reactions, the time evolution of the different states is described by a system of linear differential equations, which for a system with three dynamic states is given by

$$\frac{d}{dt}\left(\begin{array}{c} \begin{array}{c} x^{\left(1\right)} \end{array}\\ \begin{array}{c} x^{\left(2\right)}\\ x^{\left(3\right)} \end{array} \end{array}\right)=\left(\begin{array}{ccc} -\left(k_{21}+k_{31}\right) & k_{12} & k_{13}\\ k_{21} & -\left(k_{12}+k_{32}\right) & k_{23}\\ k_{31} & k_{32} & -\left(k_{13}+k_{23}\right) \end{array}\right)\left(\begin{array}{c} \begin{array}{c} x^{\left(1\right)} \end{array}\\ \begin{array}{c} x^{\left(2\right)}\\ x^{\left(3\right)} \end{array} \end{array}\right).$$

The rate constants *k*_*ij*_ describe the rates of transition from state *j* to state *i*. In general, this is expressed in matrix notation as

$$\frac{dx}{dt}=Kx,$$ (12)

where **K** is the transition rate matrix and ***x*** is the vector of the total fractions of the species. In the following, we denote the fraction of static molecules by $x_{s}^{\left(i\right)}$ and the fraction of dynamic molecules by $x_{d}^{\left(i\right)}$. Both fractions are normalized to one, i.e., $\sum_{i}x_{s}^{\left(i\right)}=1$ and $\sum_{i}x_{d}^{\left(i\right)}=1$. Hence, the total fraction of a species *x*^(*i*)^ is given by the sum of the fractions $x_{d}^{\left(i\right)}$ and $x_{s}^{\left(i\right)}$, weighted by the total fraction of dynamic molecules *p*_*d*_ as

$$x=p_{d}x_{d}+\left(1-p_{d}\right)x_{s},$$ (13)

where ***x***_*d*_ and ***x***_*s*_, are the vectors of the fractions of the dynamic and the static states, respectively, and *p*_*d*_ describes the fraction of molecules that participate in dynamic exchange.

The correlation function, *G*_*ab*_, is modeled based on the set of reaction rate constants, fluorescence properties, and the population of the static states. The general definition of the correlation function between two time-dependent signals *S*_*a*_(*t*) and $S_{b}\left(t\right)$ is given by

$$G_{ab}\left(t_{c}\right)=\frac{\left\langle S_{a}\left(t\right)S_{b}\left(t+t_{c}\right)\right\rangle }{\left\langle S_{a}\left(t\right)\right\rangle \left\langle S_{b}\left(t+t_{c}\right)\right\rangle },$$ (14)

where ⟨⋯⟩ denotes the time average over a long measurement. In the following, we assume that the signal fluctuations due to the diffusion of molecules and the conformational dynamics arise from independent sources (i.e., they are statistically independent), which is generally fulfilled for experimental systems. The contributions of diffusion and dynamics can, thus, be treated separately, and the correlation function $G_{ab}\left(t_{c}\right)$ is given in terms of the product:

$$G_{ab}\left(t_{c}\right)=\frac{1}{N}G_{\text{diff}}\left(t_{c}\right)G_{k,ab}\left(t_{c}\right)+1,$$ (15)

where *t*_*c*_ is the correlation time, *N* is the average number of molecules in the observation volume, and the factor $G_{k,ab}\left(t_{c}\right)$ describes the kinetic exchange and $G_{\text{diff}}\left(t_{c}\right)$ the diffusion of the molecules. For a 3D Gaussian detection profile, the factor $G_{\text{diff}}\left(t_{c}\right)$ is given by

$$G_{\text{diff}}\left(t_{c}\right)={\left(1+\frac{t_{c}}{t_{\text{diff}}}\right)}^{-1}{\left(1+{\left(\frac{w_{0}}{z_{0}}\right)}^{2}\frac{t_{c}}{t_{\text{diff}}}\right)}^{-1/2},$$ (16)

where *t*_diff_ is the diffusion time. The parameters *w*_0_ and *z*_0_ are the width of the focal and the axial plane of the detection volume, respectively, where the intensity decays to 1/*e*^2^ of the maximum value.

The kinetic factor $G_{k,ab}\left(t_{c}\right)$ depends on the correlation matrix $G\left(t_{c}\right)$ that describes the time evolution of the fluctuations of the species populations and on the signals ***S***_*a*_ and ***S***_*b*_ of the observed signal of the different species (given as column vectors). Then, the kinetic part of correlation can be expressed in the matrix notation as

$$G_{k,ab}\left(t_{c}\right)=\frac{{S_{a}}^{\text{T}}G\left(t_{c}\right)S_{b}}{\bar{S_{a}}\bar{S_{b}}},$$ (17)

where $\bar{S_{i}}=\left(S\cdot x\right)=\overset{n}{\underset{i=1}{\sum }}S^{\left(i\right)}x^{\left(i\right)}$ is the average of signal ***S*** over the species fractions ***x***, which corresponds to the time average of the signals under the assumption that the system is ergodic. In color-FCS, the vectors ***S***_*a*_ and ***S***_*b*_ correspond to the green and red signal intensities ***S***_*G*_ and ***S***_*R*_. In fFCS, ***S***_*a*_ and ***S***_*b*_ are the fractional fluorescence intensities of the species, obtained by weighting the signal based on the fluorescence decay using the filter functions for each species *a* and *b*, respectively.

The general solution for the correlation matrix $G\left(t_{c}\right)$ of the kinetic network in the presence of static states is given by

$$G\left(t_{c}\right)=p_{d}e^{Kt_{c}}X_{d}+\left(1-p_{d}\right)X_{s},$$ (18)

where $e^{Kt_{c}}$ describes the time evolution of the system and **X**_*d*_ and **X**_*s*_ are the diagonal matrices of the dynamic fractions ***x***_*d*_ and static fractions ***x***_*s*_, respectively (for details, see the supplementary material, Note 4). The matrix exponential $e^{Kt_{c}}$ can be solved using the eigenvalue decomposition (EVD) of the transition rate matrix,

$$K=\sum_{l=0}^{n-1}\Gamma^{\left(l\right)}\lambda^{\left(l\right)}\Rightarrow e^{Kt_{c}}=\sum_{l=0}^{n-1}\Gamma^{\left(l\right)}e^{\lambda^{\left(l\right)}t_{c}},$$ (19)

where **Γ**^(*l*)^ are the eigen-matrices and λ^(*l*)^ the eigenvalues of **K**, which relate to the measured FCS relaxation times $t_{R}^{\left(l\right)}$ by

$$\lambda^{\left(l\right)}=-1/t_{R}^{\left(l\right)}.$$ (20)

Equation (18) can, then, be expanded into a sum of exponential terms and substituted into Eq. (17) to obtain the following general expression for the kinetic correlation function in the presence of static states:

$$\begin{array}{c} G_{k,ab}\left(t_{c}\right)=1+A_{ab}^{\left(0\right)}+\overset{n-1}{\underset{l=1}{\sum }}A_{ab}^{\left(l\right)}e^{\lambda^{\left(l\right)}t_{c}},\\ A_{ab}^{\left(0\right)}=\underset{i<j}{\sum }\partial_{ab}^{\left(ij\right)}\left(x^{\left(i\right)}x^{\left(j\right)}-p_{d}x_{d}^{\left(i\right)}x_{d}^{\left(j\right)}\right),\\ A_{ab}^{\left(l\right)}=\underset{i<j}{\sum }\partial_{ab}^{\left(ij\right)}G_{ij}^{\left(l\right)},l=\left\{1\ldots n-1\right\}; \end{array}$$ (21)

where the matrix elements $G_{ij}^{\left(l\right)}$ are given by

$$G_{ij}^{\left(l\right)}=p_{d}{\left[\Gamma^{\left(l\right)}X_{d}\right]}_{ij}$$ (22)

and the factors $\partial_{ab}^{\left(ij\right)}$ describe the contrast between the species *i* and *j* and are given by

$$\partial_{ab}^{\left(ij\right)}=\frac{\left(S_{a}^{\left(i\right)}-S_{a}^{\left(j\right)}\right)}{\bar{S_{a}}}\frac{\left(S_{b}^{\left(i\right)}-S_{b}^{\left(j\right)}\right)}{\bar{S_{b}}}.$$ (23)

For details on the derivations, see the supplementary material, Note 4.

To obtain the correlation functions, we need to define the correlated signal vectors ***S***_*a*_ and ***S***_*b*_. In color-FCS, these signals are the detected “green” (donor) and “red” (acceptor) signal intensities ***S***_*G*_ and ***S***_*R*_:

$$\begin{array}{c} S_{a},S_{b}=\left\{\begin{array}{c} q_{G}=Q_{0}\left(1-E\right),\;\\ q_{R}=Q_{0}\left(\gamma E+\alpha \left(1-E\right)\right),\; \end{array}\right. \end{array}$$ (24)

where ***E*** is a vector whose elements correspond to the FRET efficiencies of the fluorescence species, *Q*_0_ is the molecular brightness of the donor in the absence of FRET, *α* is the crosstalk from the donor fluorophore into the red detection channel. *γ* is a combined correction parameter relating the donor and acceptor fluorescence quantum yield and the detection efficiencies of the green and red channels.^51–53^ For simplicity, we assume that the crosstalk *α* of the donor fluorescence into the red detection channel of the acceptor is zero, that the *γ*-factor is one, and that there is no background signal. In the expressions of the normalized correlation function, the scaling factor *Q*_0_ cancels out. For the simplest case of a two-state dynamic system in the presence of static states, we then obtain the general expression for the kinetic correlation function,

$$\begin{array}{ll} G_{k,ab}\left(t_{c}\right) & =1+\partial_{ab}^{\left(12\right)}\left(x^{\left(1\right)}\left(1-x^{\left(1\right)}\right)\right.\\ & \;\left.+\;p_{d}x_{d}^{\left(1\right)}\left(1-x_{d}^{\left(1\right)}\right)\left(e^{-\left(k_{12}+k_{21}\right)t_{c}}-1\right)\right), \end{array}$$ (25)

where the pre-factor $\partial_{ab}^{\left(12\right)}$ depends on the channels that are correlated,

$$\partial_{ab}^{\left(12\right)}=\left\{\begin{array}{cc} {\left(E^{\left(1\right)}-E^{\left(2\right)}\right)}^{2}/{\left(1-\bar{E}\right)}^{2},\; & ab=GG,\\ {\left(E^{\left(1\right)}-E^{\left(2\right)}\right)}^{2}/{\bar{E}}^{2},\; & ab=RR,\\ -{\left(E^{\left(1\right)}-E^{\left(2\right)}\right)}^{2}/\left(\bar{E}\left(1-\bar{E}\right)\right),\; & ab=RG,GR, \end{array}\right.$$ (26)

where the average FRET efficiency $\bar{E}$ is given by the average over the total species fractions *x*^(*i*)^. The complete derivation of the analytical form of the correlation function for two- and three-state systems is outlined in the supplementary material, Note 4. Corresponding expressions had previously been obtained for two-state dynamic systems in the presence of a third static state.^15^

#### 1. Ambiguities in color-FCS

Before applying the formalism derived in Sec. IV A for the quantitative analysis of the simulated datasets, we emphasize why the combination of FCS and TCSPC is needed. Color-FCS (or FRET-FCS) analysis is generally underdetermined as there are more model parameters than experimentally accessible parameters. Thus, it is required that the FRET efficiency of at least one of the two states is known, but better results are obtained if both FRET efficiencies are constrained.^13^ The origin of this ambiguity is outlined in the following.

For a purely dynamic two-state system, i.e., in the absence of static molecules, the expression for the correlation function given in Eq. (25) simplifies to

$$G_{k,ab}\left(t_{c}\right)=1+\partial_{ab}^{\left(12\right)}x_{d}^{\left(1\right)}\left(1-x_{d}^{\left(1\right)}\right)e^{-\left(k_{12}+k_{21}\right)t_{c}}.$$ (27)

In an experiment, we measure two autocorrelation functions (GG and RR) and two cross correlation functions (GR and RG). The cross correlation functions contain identical information, and the time constant of the exponential term is shared between all correlation functions. Thus, we determine three correlation amplitudes, $\partial_{ab}^{\left(12\right)}x_{d}^{\left(1\right)}\left(1-x_{d}^{\left(1\right)}\right)$, and the decay rate of the exponential term, *k*_12_ + *k*_21_. As is evident from Eq. (26), however, the cross correlation amplitude relates to the autocorrelation amplitudes by

$$\partial_{GR}^{\left(12\right)}=-\sqrt{\partial_{GG}^{\left(12\right)}\partial_{RR}^{\left(12\right)}},$$ (28)

and it contains no independent information. The system is, thus, underdetermined, as we have access to only three experimental observables compared to the four parameters of the model (*E*^(1)^, *E*^(2)^, *k*_12_, and *k*_21_). The ambiguity between the model parameters takes a complex form and is illustrated in the supplementary material, Note 5. This ambiguity is resolved if the FRET efficiencies of the states are known from single-molecule FRET efficiency histograms or fluorescence decay analysis. In the following, we explore the combination of FCS with TCSPC to restrain the FRET efficiencies of the states using the information provided by the fluorescence decays, which enables quantitative analysis of the kinetics by FCS.

#### 2. Joint analysis of fluorescence decays and FCS

To unambiguously resolve all contributing states and their exchange rates, we combine the information provided by FCS and TCSPC and optimize all model parameters globally. While FCS is sensitive to the relaxation rate constants, TCSPC informs the FRET efficiencies and the total species fractions. Thus, the two methods provide orthogonal information that defines the FRET efficiencies of the states and the transition rate matrix. The global analysis is also expected to stabilize the optimization algorithm and reduce the uncertainty of the model parameters.

The donor fluorescence decay of the FRET sample $f_{D|D}^{\left(DA\right)}\left(t\right)$ depends on the FRET efficiencies of the species and the associated total species fractions *x*^(*i*)^. We assume that the time scale of fluorescence and the time scale of dynamics are decoupled. In other words, the fluorescence lifetime is much shorter than the relaxation time of the kinetic processes. Therefore, the fluorescence decay of the ensemble of molecules can be described by the total fractions of the FRET species *x*^(*i*)^ and their FRET efficiencies *E*^(*i*)^:

$$f_{D|D}^{\left(DA\right)}\left(t\right)=\sum x^{\left(i\right)}\;\exp\left(-\frac{t}{\tau_{D\left(0\right)}\left(1-E^{\left(i\right)}\right)}\right),$$

where

$$x^{\left(i\right)}=\left(1-p_{d}\right)x_{s}^{\left(i\right)}+p_{d}x_{d}^{\left(i\right)}.$$ (29)

To optimize the model parameters, we define a global goodness-of-fit function, $\chi_{\text{global}}^{2}$, as the sum of the squared weighted deviations for TCSPC, $\chi_{\mathrm{TCSPC}}^{2}$, and FCS, $\chi_{\mathrm{FCS}}^{2}$,

$$\begin{array}{ll} \chi_{\text{global}}^{2}=\chi_{\mathrm{TCSPC}}^{2}+\chi_{\mathrm{FCS}}^{2} & =\underset{t}{\sum }{\left(\frac{f_{D|D}^{\left(DA\right),\exp.}\left(t\right)-f_{D|D}^{\left(DA\right)}\left(t\right)}{\sigma_{\mathrm{TCSPC}}\left(t\right)}\right)}^{2}\\ & \;+\;\underset{a,b}{\sum }\underset{t_{c}}{\sum }{\left(\frac{G_{ab}^{\exp\cdot }\left(t_{c}\right)-G_{ab}\left(t_{c}\right)}{\sigma_{\mathrm{FCS}}\left(t_{c}\right)}\right)}^{2}. \end{array}$$ (30)

Here, the weighting factors, *σ*_*TCSPC*_ and *σ*_*FCS*_, account for the nonuniform measurement uncertainty in the data. For TCSPC, the weighting factor is estimated based on the experimental counts under the assumption of Poissonian counting statistics as $\sigma_{\mathrm{TCSPC}}\left(t\right)=\sqrt{f_{D|D}^{\left(DA\right),\exp.}\left(t\right)}$. For FCS, the weights are estimated based on the recorded data as described in Kask *et al.*^54^ Overall, one experimental fluorescence decay $f_{D|D}^{\left(DA\right)}\left(t\right)$ and four correlation curves [*G*_*GG*_(*t*_*c*_), *G*_*GR*_(*t*_*c*_), $G_{RG}\left(t_{c}\right)$, and $G_{RR}\left(t_{c}\right)$] contribute to $\chi_{\text{global}}^{2}$.

### B. Analysis of simulations

#### 1. Analysis of three-state kinetic networks

**FIG. 5.**
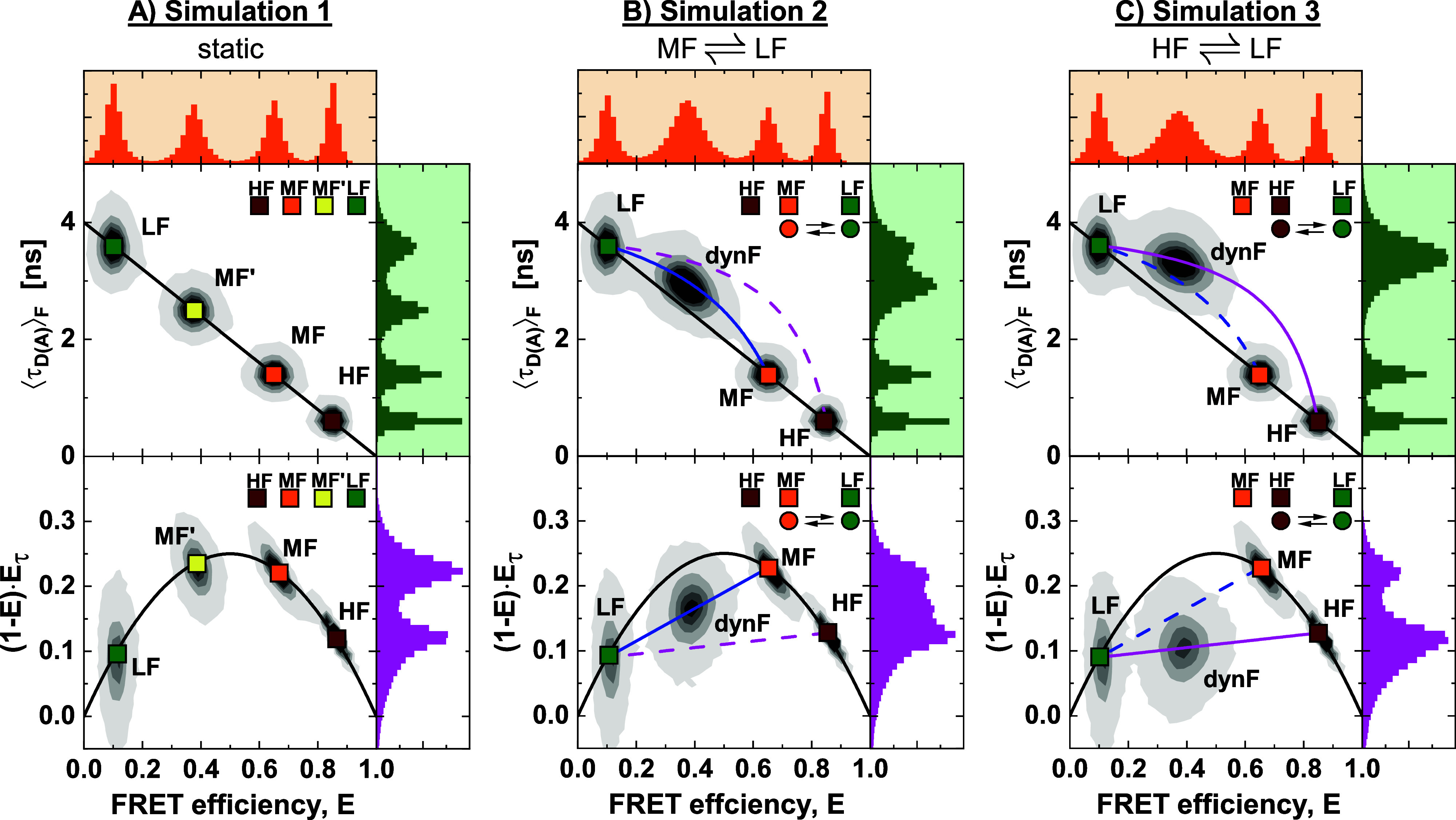
Simulations of heterogeneous mixtures of static and dynamic molecules. Three different simulations were performed and are displayed in the (*E*, ${\left\langle \tau_{D\left(A\right)}\right\rangle }_{F}$) (top) and moment representations (bottom). (a) Simulation 1: A mixture of four static FRET species with high (HF), medium (MF; MF′), and low (LF) FRET efficiency, resulting in a 1D FRET efficiency histogram with four distinct peaks. (b) Simulation 2: A mixture of three static species and one dynamic FRET population fluctuating between MF and LF species with exchange rate constants *k*_*MF*→*LF*_ = *k*_*LF*→*MF*_ = 5 ms^−1^. This exchange results in a heterogeneous dynamic population (dynF) with an average FRET efficiency equal to that of the MF′ species of simulation 1. (c) Simulation 3: A mixture of three static species and one dynamic FRET population fluctuating between an HF and an LF species. The exchange rate constants are *k*_*HF*→*LF*_ = 6.3 ms^−1^ and *k*_*LF*→*HF*_ = 3.7 ms^−1^. The black line corresponds to a static FRET-line. The magenta and blue lines correspond to dynamic FRET-lines describing HF/LF and MF/LF mixtures. Solid lines indicate the simulated exchange, while dashed lines correspond to the kinetic transition that was not considered in the simulation. The set of simulation parameters is given in the  supplementary material, Tables 2 and 3.

To test the global analysis framework for the analysis of multi-state systems, we simulated a series of experiments. We consider a heterogeneous mixture of various static and dynamic FRET species (Fig. 5 and supplementary material, Tables 2 and 3).

No linker dynamics were included in the simulations. First, we consider four distinct static species with low, medium, and high FRET efficiency [LF, MF, MF′, and HF species, respectively, Fig. 5(a) and supplementary material, Fig. 9]. As expected, the four populations in the two-dimensional histograms lie on the static FRET-line, and no indication for conformational dynamics is seen. As defined in Fig. 1, we then simulated heterogeneous mixtures of three static FRET species with additional two dynamic FRET species in dynamic exchange [Figs. 5(b) and 5(c)]. The kinetic rates were chosen such that the resulting dynamic population has an average FRET efficiency identical to that of the MF′ population in the static mixture, resulting in almost indistinguishable one-dimensional FRET efficiency histograms for the three simulations. By overlaying the dynamic FRET-lines connecting the static species, the interconverting species of the dynamic population can be assigned (solid lines). Dynamic FRET-lines of species that are not in dynamic exchange do not intersect with the dynamic population (dashed lines). Moreover, as the dynamic populations are positioned directly on the limiting binary dynamic FRET-lines, we can exclude the possibility of ternary exchange between all three species, which would instead result in a population positioned within the area defined by the three limiting lines (compare Secs. 3.F and 3.G of Paper I^28^). The graphical analysis by FRET-lines, thus, provides a simple approach to determine the kinetic connectivity of the network. For the moment representation (Fig. 5, bottom), dynamic FRET-lines can be drawn as simple lines. While the equilibrium constant may be extracted from the plots given the fast dynamics in these examples (as described in Sec. III), additional information is required to quantify the exchange rates of the kinetic network.

**FIG. 6.**
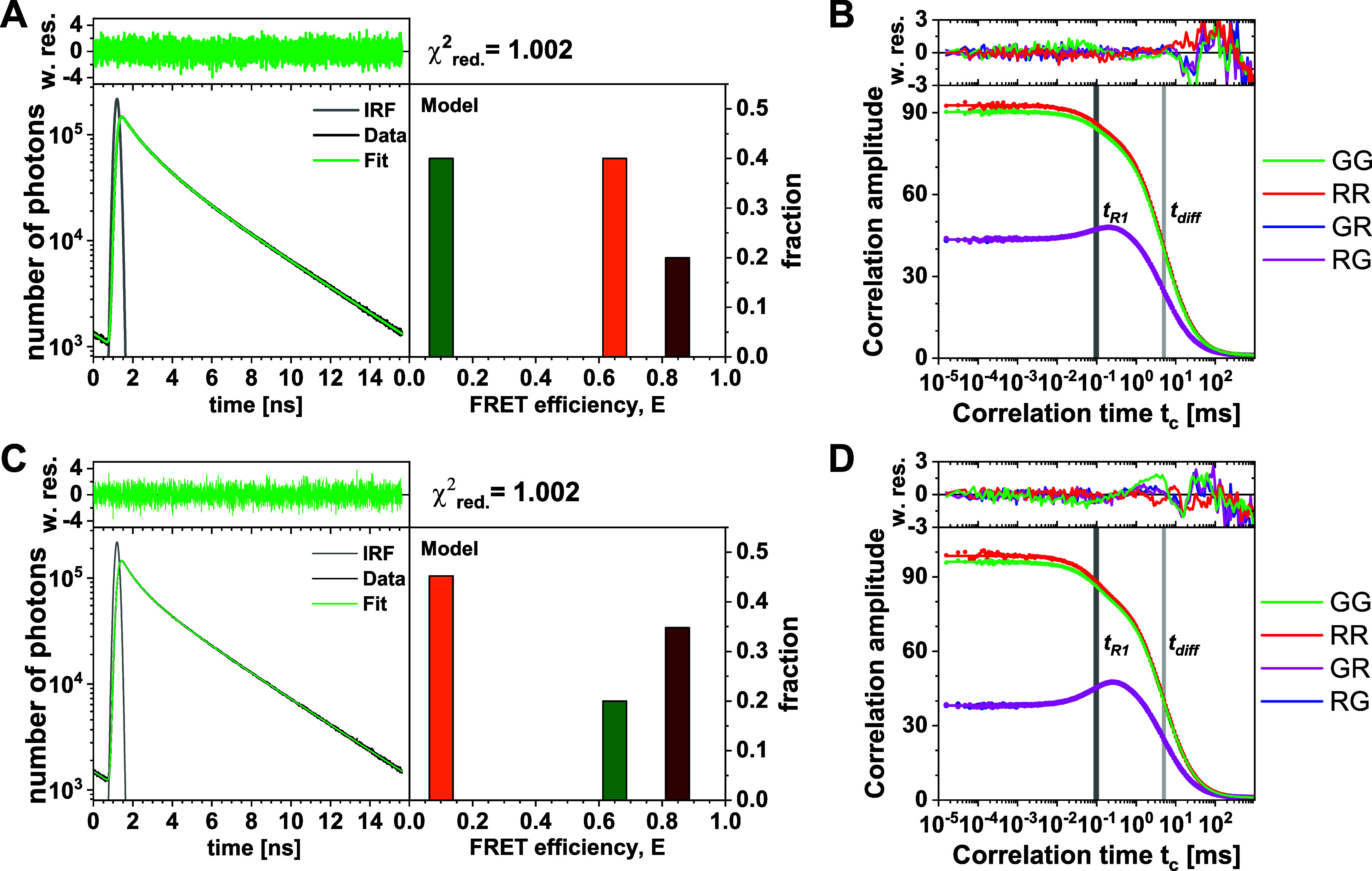
Global analysis of simulation 2 (a) and (b) and 3 (c) and (d). (a) and (c) Ensemble fluorescence decays of the donor fluorophore (left) and recovered FRET efficiency components (right). (b) and (d) Color-FCS autocorrelation and cross correlation functions reveal a single relaxation time *t*_*R*1_ = 100 *µ*s for both simulations. The kinetics are superimposed on the diffusion term of the correlation function with a diffusion time *t*_diff_ = 5 ms. Weighted residuals (w.res.) of the fits are given above. The simulation parameters are given in the supplementary material, Tables 1 and 2.

In the following, we apply the global analysis of FCS and TCSPC to the simulated datasets shown in Fig. 5. For the simulation of four static species [Fig. 5(a)], the absence of conformational dynamics is confirmed by the absence of a kinetic contribution in the FCS curves (supplementary material, Fig. 9). For the simulations with dynamics between two species [Figs. 5(b) and 5(c)], static and dynamic species are indistinguishable in analyzing the fluorescence decays, which are well described by a three-state model [Figs. 6(a) and 6(c)].

The green/red cross correlation curves show a pronounced anti-correlation, which reveals conformational dynamics [Figs. 6(b) and 6(d)]. In the analysis of the correlation curves, a single relaxation time of ∼100 *µ*s is sufficient to describe the data in both cases. This implies that only two of the three species are in dynamic exchange, consistent with the graphical analysis performed by FRET-lines. We, thus, consider three possible kinetic schemes that differ in the assignment of the purely static species:

$$\begin{array}{c} MF,HF⇌LF,\\ HF,MF⇌LF,\\ LF,HF⇌MF. \end{array}$$ (31)

In this notation, MF, HF ⇌ LF refers to the scheme where the HF and the LF species are exchanging, and the MF species is purely static.

To distinguish among the possible kinetic schemes in Eq. (31) and to quantify the microscopic parameters, we use the global analysis of FCS and TCSPC. In total, there are nine model parameters: three FRET efficiencies *E*^(*i*)^ for the FRET species HF, MF, and LF; two exchange rate constants *k*_*ij*_ to describe the exchange among the two dynamic species; two independent fractions ${x_{s}}^{\left(i\right)}$ of the static species (the third is determined by the other two); and the probability that a molecule is in a dynamic state *p*_*d*_. These *microscopic parameters* define the experimental observables, such as the slope of the fluorescence decays or the amplitudes and relaxation timescales of the FCS curves. The fluorescence decays are fully described by the FRET efficiencies *E*^(*i*)^ and total species fractions *x*^(*i*)^ [see Eq. (29)], while the relation between the microscopic parameters and the amplitudes of the FCS curves is more complex. In the description of the FCS model function, we had split the contributions of the FRET efficiencies of the different states from the quantities that depend only on the parameters of the kinetic network [see Eq. (25)]. The relaxation time of the kinetic amplitude is given by the inverse of the sum of the exchange rates, $t_{R}={\left(k_{12}+k_{21}\right)}^{-1}$. The amplitudes of the auto- and cross correlation curves depend mainly on the total species fractions and FRET efficiencies, which are determined from the information provided by TCSPC. As described in detail in the supplementary material, Note 6, the only new information obtained from the amplitudes of the FCS curves is the relative amplitude of the kinetic term, i.e., the pre-exponential factor in Eq. (25) given by $p_{d}x_{d}^{\left(1\right)}x_{d}^{\left(2\right)}$. Thus, only seven parameters are available from the experiment: the FRET efficiencies and static fractions obtained from TCSPC and the sum of the rates and the product $p_{d}x_{d}^{\left(1\right)}x_{d}^{\left(2\right)}$ from the FCS curves. The system is, hence, inherently underdetermined, and ambiguity is expected between the fraction of dynamic molecules *p*_*d*_ and the exchange rates *k*_*ij*_ that define the dynamic fractions *x*_*d*_,

$$p_{d}x_{d}^{\left(1\right)}x_{d}^{\left(2\right)}\propto p_{d}k_{12}k_{21}=p_{d}k_{12}\left(t_{R}^{-1}-k_{12}\right).$$ (32)

#### 2. Resolving complex kinetic networks using the global analysis framework

**FIG. 7.**
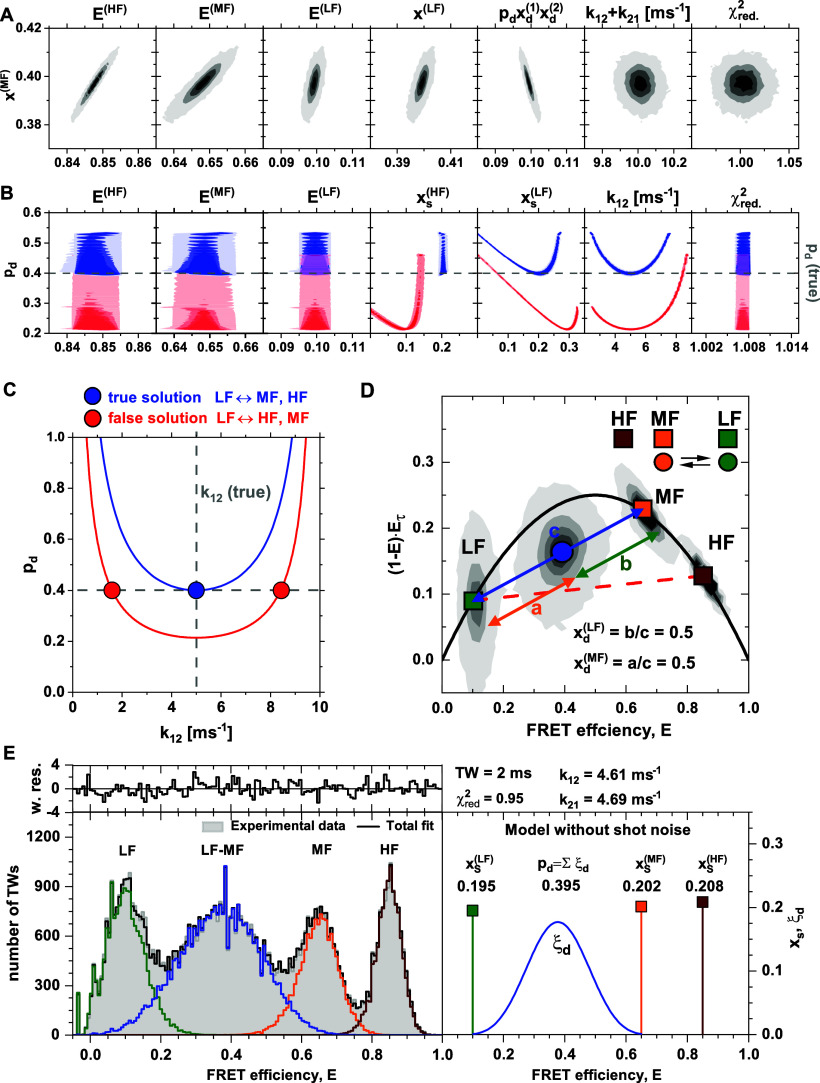
Global analysis of simulation 2. (a) Pairwise distributions of the independent experimental parameters for the total fraction of molecules in the medium-FRET species x^(MF)^ determined by Markov chain Monte Carlo sampling. The parameters show narrow distributions centered at the ideal values due to the ideal signal-to-noise ratio in the simulations. (b) Pairwise distributions of the microscopic model parameters and $\chi_{r}^{2}$ for the total fraction of dynamic molecules p_d_. Due to the ambiguity of the experimental parameters, two different kinetic schemes (blue: LF ⇌ MF, red: LF ⇌ HF) are consistent with the data as indicated by the identical $\chi_{r}^{2}$ (right). Each of these two kinetic schemes is additionally consistent with a range of model parameters. If the correct kinetic scheme (blue: LF ⇌ MF) and the total fraction of dynamic molecules are known, the unique solution can be identified (gray dashed line). (c) Ambiguities arising in the global analysis. The first ambiguity arises from the fact that the system is inherently underdetermined, preventing both p_d_ and k_12_ to be resolved at the same time (dashed lines). Additionally, due to the ambiguity of the FCS amplitudes, different kinetic schemes can be compatible with the data, resulting in two branches of the solution space in the given case (red: false solution, blue: true solution). (d) A graphical analysis by FRET-lines resolves the connectivity within the kinetic network. The position of the dynamic population on the dynamic FRET-line between the species LF and MF shows that the kinetics occur between the LF and MF species, while the HF species is static [blue line in (c)]. The dynamic FRET-line for the competing solution [red line (c)] is shown as a red dashed line. To resolve the ambiguity between the parameters p_d_ and k_12_ in the general case of k_12_ ≠ k_21_, it is additionally required to determine the dynamic species fractions $x_{d}^{\left(i\right)}$. In the case of fast dynamics as given here, this parameter can be estimated from the position of the dynamic population along the dynamic FRET-lines. The quantities *a*, *b,* and *c* represent the length of the vectors connecting the LF species and the dynamic population (orange), the MF species and the dynamic population (green), and the LF and MF species (blue), respectively. For slower dynamics, the mode of the population deviates from the true mean, and the procedure described in Sec. III should be applied. (e) A dynamic photon distribution analysis (PDA) of the simulated data identifies static and dynamic species and quantifies the exchange rates (green: LF, orange: MF, dark red: HF, blue: dynamic population from LF ⇌ MF exchange). A global analysis of the FRET efficiency histograms obtained for time windows (TW) of 1–3 ms was performed to improve the sensitivity of the analysis to dynamics^8^ (see the supplementary material, Fig. 10). The displayed FRET efficiency histogram (gray) corresponds to a time window of 2 ms. The estimated fractions of the static and dynamic species are shown on the right.

To test this prediction, we sampled the probability distribution of the parameters for the possible kinetic networks given in Eq. (31) using a Markov chain Monte Carlo (MCMC) approach. The resulting distributions are shown as two-dimensional contour plots in Fig. 7(a). Indeed, the experimentally accessible parameters show defined, narrow distributions due to the high signal-to-noise ratio of the simulated data.

However, the distributions of the microscopic parameters exhibit the expected ambiguity [Fig. 7(b)], which arises because only the product $p_{d}x_{d}^{\left(1\right)}x_{d}^{\left(2\right)}$ can be quantified. Additionally, a second ambiguity arises between the different realizations of the kinetic network in Eq. (31). By considering the permutations of the kinetic scheme, different assignments of the FRET efficiencies to the static and dynamic species can result in identical FCS amplitudes, splitting the solution space into two branches (see the supplementary material, Note 6). For the given example of simulation 2, two competing solutions exist between the schemes LF ⇌ MF, HF, and LF ⇌ HF, MF [colored blue and red in Fig. 7(b)]. In contrast, the third permutation results in nonphysical solutions for the microscopic parameters. These two solutions are indistinguishable in the analysis framework, as is evident from the identical reduced $\chi_{\text{global}}^{2}$ [Fig. 7(b)]. The observed ambiguities can be described analytically based on the analytical model functions if the actual parameters are known. The resulting relation between the parameters *p*_*d*_ and *k*_12_ is shown in Fig. 7(c) and described in detail in the supplementary material, Note 6.

The question remains how these ambiguities can be resolved. To decide between the two branches corresponding to the different state assignments, FRET-lines provide the required information by identifying the kinetic connectivity of thenetwork. From the dynamic FRET-lines, we had identified LF ⇌ MF, HF as the true solution [Figs. 5(b) and 7(d)], allowing us to eliminate the competing solution LF ⇌ HF, MF. To resolve the ambiguity arising from the presence of purely static states [Eq. (32)], it is required to determine either the total fraction of dynamic molecules *p*_*d*_ or the rate constant *k*_12_ [see dashed lines in Fig. 7(c)]. For fast dynamics, *p*_*d*_ is directly accessible from the two-dimensional histograms as the fraction of molecules in the dynamic population that deviates from the static FRET-line. However, this approach does not apply to slower dynamics due to pseudo-static species on the static FRET-line. In this case, a photon distribution analysis can be applied to recover *p*_*d*_, which will be discussed below.

In the given example, the knowledge of *p*_*d*_ resolves the ambiguity because the exchange rates were chosen equal (*k*_12_ = *k*_21_ = 5 ms^−1^). However, an ambiguity remains for the general case of *k*_12_ ≠ *k*_21_ regarding the assignment of the exchange rates to the dynamic species (see the supplementary material, Note 6, for details). Knowledge of the rate *k*_12_ (or *k*_21_) resolves the ambiguity in the analysis in all cases. To define the rate *k*_12_, it is sufficient to know the FCS relaxation time *t*_*R*_ and the dynamic fraction $x_{d}^{\left(1\right)}$. As we have shown in Sec. III, this information may be obtained from the mode of the dynamic distribution for intermediate to fast dynamics. For the fast dynamics of the system discussed here, one may also estimate the dynamic fraction $x_{d}^{\left(1\right)}$ directly from a graphical analysis in the moment representation from the position of the dynamic population along the dynamic FRET-line connecting the LF and MF species [Fig. 7(d)]. The dynamic population is positioned at the center of the line, and the resulting dynamic fractions are $x_{d}^{\left(LF\right)}=x_{d}^{\left(MF\right)}=0.5$.

The fraction of dynamic molecules *p*_*d*_ may also be obtained from a detailed analysis of the FRET efficiency histogram by a dynamic photon distribution analysis (PDA) using a combination of static and dynamic populations [Fig. 7(e) and supplementary material, Fig. 10].^8^ The analysis yields the correct fraction of dynamic molecules of *p*_*d*_ = 0.395. If the correct model (LF ⇌ MF) is known *a priori*, PDA also recovers exchange rates that are close to the ground truth ($k_{12}^{\text{PDA}}=4.61$ ms^−1^, $k_{21}^{\text{PDA}}=4.69$ ms^−1^). While PDA clearly rules out a static model [supplementary material, Fig. 11(a)], it should be noted that, in the given case, PDA cannot distinguish LF ⇌ MF from LF ⇌ HF exchange [supplementary material, Fig. 11(b)]. A PDA with the wrong model (LF ⇌ HF) achieves a similar fit quality and yields an exchange rate constant of $k_{12}^{\text{PDA}}=10.0$ ms^−1^, $k_{21}^{\text{PDA}}=6.08$ ms^−1^ (supplementary material, Table 4). Interestingly, even though the wrong model was used, PDA correctly quantified the fraction of dynamic molecules as *p*_*d*_ = 0.397. The ambiguity between the two models can be resolved as before by inferring the correct connectivity from the two-dimensional histograms using FRET-lines [Fig. 7(d)]. Note that the reason why the LF ⇌ HF model achieves a similar fit quality compared to the correct LF ⇌ MF is that the exchange is fast compared to integration time, resulting in almost complete averaging even during the shorted time window of 1 ms. The single dynamically averaged population can then be approximated by fast mixing between the LF species and either the MF or HF species, and the rates are adjusted accordingly to match the observed average FRET efficiency of the dynamic population. In contrast, a model assuming exchange between MF and HF species is incapable of describing the data in PDA (data not shown). The observed ambiguity in PDA would not occur in the case of slower exchange rates.

##### a. Applicability and limitations of the presented approach.

In summary, we have demonstrated that, even for simple two-state kinetic networks in the presence of a background of static molecules, a global analysis of TCSPC and FCS provides ambiguous solutions. These ambiguities can partially be resolved using FRET-lines to eliminate models that are incompatible with the data. Additionally, it is required to know either the total fraction of dynamic molecules *p*_*d*_ or the equilibrium constant of the dynamic process to fully determine the microscopic parameters.

So far, we have applied the global analysis workflow to simulated data only. The presented simulations present ideal scenarios with well-separated species and dynamic relaxation times that do not overlap with the timescale of diffusion. Arguably, the most challenging experimental situation would be given by the existence of degenerate species, i.e., conformational states with identical FRET efficiency but different exchange rates (e.g., pseudo-static and dynamic states in Fig. 1), which would be indistinguishable in the TCSPC analysis. However, when the exchange rates differ substantially, degenerate states would still be detectable from their kinetic features, i.e., the number of relaxation times in the FCS curves. Hence, while the accuracy of the analysis would be reduced, the combination of structural and kinetic information in the global analysis workflow should still allow for a quantitative analysis even in this challenging case. For the case that the relaxation times overlap with the timescale of diffusion, the global analysis of the auto- and cross correlation functions should still provide a clear identification of the contribution of conformational dynamics from the distinct anti-correlated signature in the donor–acceptor cross correlation function. For clarity, we have also assumed ideal experimental conditions in this work with respect to high counting statistics for the number of detected molecules and the number of photons per single-molecule event. As a result, the reported uncertainties for the inferred model parameters are exceptionally low [Fig. 7(a)], and higher uncertainties are expected for real experiments where generally a lower number of bursts (<10^4^) and photons per burst (∼100) are available. Furthermore, while we did not consider the effect of background signal and other experimental imperfections such as spectral crosstalk of the donor into the acceptor detection channel or direct excitation of the acceptor by the donor excitation laser, corrections for these experimental complications are easily applied using established protocols for intensity-based^53^ or time-resolved^18^ experiments. Additional experimental complications derive from the photophysics of the dyes due to photobleaching and blinking (e.g., due to the population of triplet or radical-ion dark states). Since these phenomena affect the brightness of the fluorophores, they will contribute to the FCS curves and distort the species fractions estimated from the TCPSC analysis. In this case, careful controls are necessary to ensure that the detected dynamics are biologically relevant (reviewed in detail in Ref. 1). To limit the complications due to photobleaching and slow blinking, we recommend a strict burst selection using established criteria.^37,55,56^ For TCSPC and color-FCS, a sub-ensemble analysis is advised to limit the detrimental contribution of donor-only signal.^13,57^

**FIG. 8.**
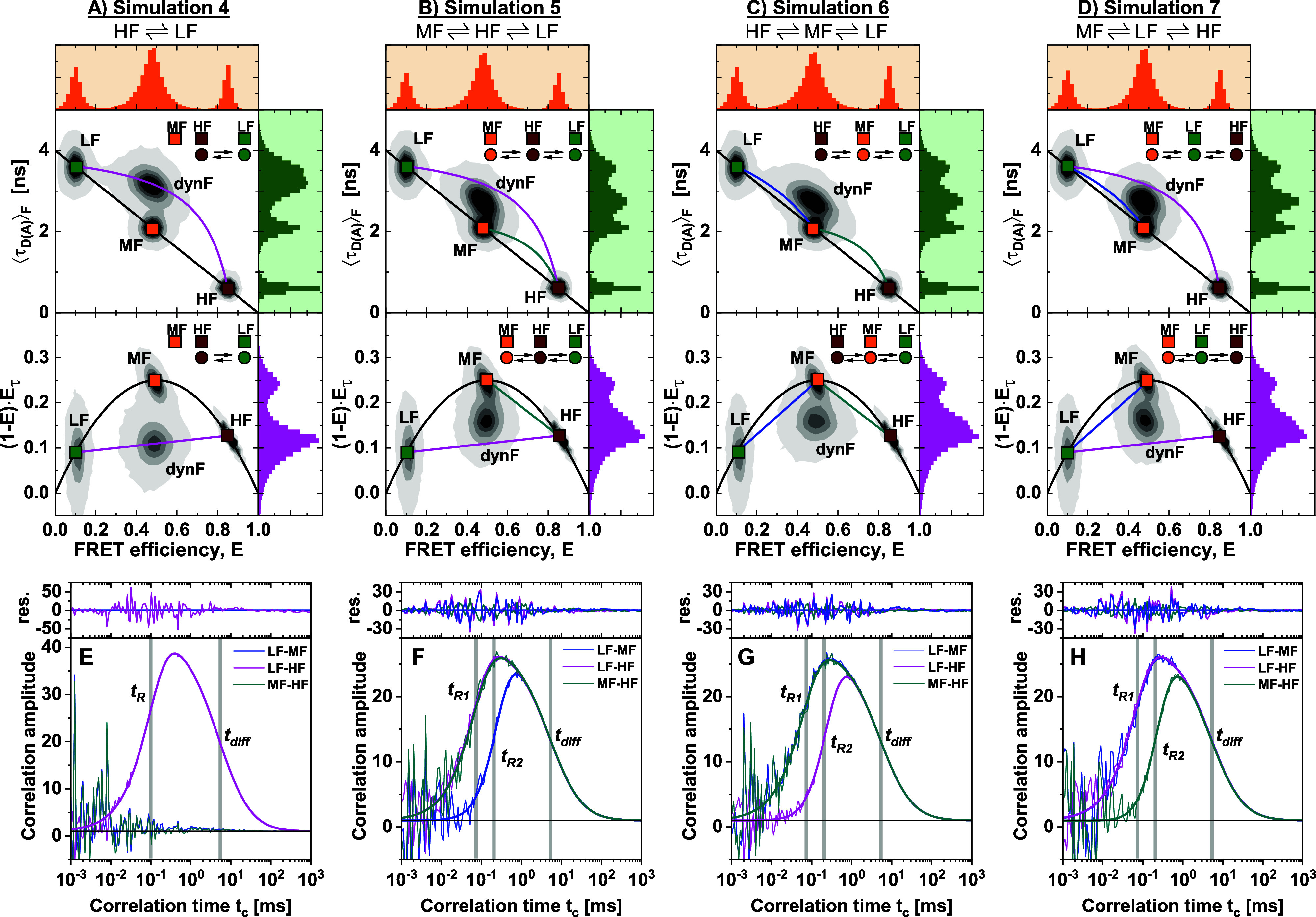
Simulations of heterogeneous mixtures of static and dynamic molecules involving three-state kinetic networks. Four different simulations were performed and are displayed in the (*E*, ${\left\langle \tau_{D\left(A\right)}\right\rangle }_{F}$) (top) and moment (bottom) representations. (a) Simulation 4: A mixture of three static FRET species with high (HF), medium (MF), and low (LF) FRET efficiency and one dynamic species (dynF) with binary dynamics between the LF and HF species with exchange rate constants *k*_*HF*→*LF*_ = *k*_*LF*→*HF*_ = 5 ms^−1^. (b)–(d) Simulations 5–7: Mixture of three static species (LF, MF, HF) and one dynamic FRET population fluctuating between all three species with exchange rate constants *k*_*i*→*j*_ = 5 ms^−1^ in linear kinetic schemes with different connectivity. The fast exchange results in a defined heterogeneous dynamic population (dynF) with equal average FRET efficiency for the three different kinetic networks. The black line corresponds to a static FRET-line. The magenta and blue lines correspond to dynamic FRET-lines describing HF/LF and MF/LF mixtures. Solid lines indicate the simulated exchange, while dashed lines correspond to the kinetic transition that was not considered in the simulation. The set of simulation parameters is given in the supplementary material, Tables 2 and 3. (e)–(h) Corresponding species cross correlation functions between the three species LF, MF, and HF for the different simulations. For the binary exchange (a) and (e), a positive cross correlation signal is obtained only for the exchanging species (LF–HF). For the linear three-state kinetic networks, the delayed exchange is detected between species that show no direct connectivity, while connected species exchange faster. The correlation curves are fitted to a kinetic model involving one (e) or two (f)–(h) relaxation times *t*_*R*_ and a diffusion model with a global diffusion time *t*_diff_. Weighted residuals of the fits are given above. The corresponding autocorrelation curves are given in the supplementary material, Fig. 12.

#### 3. Resolving complex kinetic networks by filtered-FCS

In Sec. IV B 2, we showed how binary kinetic exchange in the presence of a background of static molecules could be resolved by integrative analysis of fluorescence decays, FCS curves, and FRET-lines. To provide more challenging test cases, we performed a series of simulations with an exchange between three species in a linear reaction scheme. We compare binary exchange HF ⇌ LF Fig. 8(a) possible kinetic schemes with three dynamic species: MF ⇌ HF ⇌ LF, HF ⇌ MF ⇌ LF, and MF ⇌ LF ⇌ HF [Figs. 8(b)–8(d)]. Due to the fast kinetic exchange, complete averaging is observed for the dynamic population (dynF). The exchange rates were chosen such that the FRET efficiency *E* and fluorescence-weighted average lifetime ${\left\langle \tau_{D\left(A\right)}\right\rangle }_{F}$ are identical for the dynamic population. Consequentially, the four scenarios are in principle indistinguishable for the two-dimensional histograms of ${\left\langle \tau_{D\left(A\right)}\right\rangle }_{F}$ vs *E* (top row) or the moment representation (bottom row). While the corresponding dynamic FRET-lines indicate the correct kinetic pathways in Figs. 8(b)–8(d), the fast exchange renders it impossible to resolve the kinetic network in this case. However, the position of the dynamic population between the limiting FRET-lines of the binary exchanges is a clear indication for a three-state exchange. As described in Sec. III, the equilibrium fractions of the contributing species can be determined by a graphical analysis in this case.

To analyze the kinetics in the complex scenario of fast three-state dynamics, we apply filtered fluorescence correlation spectroscopy (fFCS).^13,26^ fFCS exploits the information contained in the fluorescence decays to increase the selectivity and contrast of the correlation functions. By characterizing the different species in the mixture by their fluorescence decay patterns, filters are constructed that allow one to separate the contributions of the different species to the correlation function using statistical weights. Due to the orthogonality of the filters, the resulting correlation functions are species-specific. Thus, it is possible to resolve the binary exchange between different species even in complex mixtures and obtain information on the respective relaxation times *t*_*R*_. A distinct advantage of fFCS is that the number of correlation curves increases to the square of the number of contributing species. In contrast, for cFCS, the number of correlation functions is limited by the number of color detection channels (four for two-color detection). However, fFCS requires prior knowledge of the number of species and depends crucially on the quality of the filters, which require precise knowledge of the fluorescent properties of each species. Given these prerequisites, fFCS can reveal the kinetic connectivity and quantify the exchange rate constant of the kinetic network.

To show the potential of fFCS, we return to the previous test cases of binary exchange between two species in the kinetic scheme MF, LF ⇌ HF [Fig. 8(a)]. For this simulation, the two-dimensional histogram reveals four peaks. Three of the four peaks (HF, MF, and LF) are located on the static FRET-line, corresponding to molecules with constant fluorescence properties during the observation time. The dynamic population (dynF) is positioned on the dynamic FRET-line describing the exchange between the LF and HF populations and reveals the dynamic exchange between these species. Only the cross correlation function between the LF and HF species shows a positive signal [Fig. 8(e)], while the corresponding species autocorrelation functions reveal a positive correlation term that matches the timescale of the rise of the cross correlation function (supplementary material, Fig. 12). From a global analysis of the species auto- and cross correlation functions, we obtain a single relaxation time, *t*_*R*_, which relates to the exchange rates by $t_{R}=1/\left(k_{LF\to HF}+k_{HF\to LF}\right)$. The cross correlation functions that interrogate the MF ⇌ HF or LF ⇌ MF transitions, on the other hand, show no amplitude, proving that there is no exchange between these species.

For fast-exchanging processes, it is not possible to resolve the kinetic network from visual inspection of the two-dimensional histograms [Figs. 8(b) and 8(c)]. To address this problem, we computed all possible cross correlation functions using specific filters for the three species HF, MF, and LF [Figs. 8(f) and 8(g)]. There is no direct connection between the LF and MF species for the linear kinetic scheme MF ⇌ HF ⇌ LF [Fig. 8(f)]. Correspondingly, the exchange between these species is delayed compared to the direct transitions between the MF/HF and HF/LF species, as is evident from the delayed rise of the LF–MF species cross correlation function (sCCF) compared to the HF–LF and the HF–MF sCCF. Identical observations are made for the kinetic networks LF ⇌ MF ⇌ HF and MF ⇌ LF ⇌ HF [Figs. 8(g) and 8(h)], showing a delayed rise of the cross correlation for the indirect pathway. We can, thus, obtain qualitative information about the connectivity in the network through the relaxation times of the species cross correlation functions and identify dominant pathways in the network. However, it is impossible to exclude the possibility of exchange between species from the delayed exchange alone. Similar results would, for example, be obtained for fully connected kinetic networks with a slow exchange between two species. The number of relaxation times required to describe the data provides information about the minimum number of states of the network. Since the relaxation times correspond to the inverse of the nonzero eigenvalues of the transition rate matrix, a kinetic network involving *N* states shows *N* − 1 relaxation times in the correlation functions.

In summary, fFCS enables the direct interrogation of transitions between distinct species and allows us to recover the relaxation times and the connectivity within the kinetic network. Indirect transitions show a delayed rise of the SCCF compared to direct transitions. Nonunique solutions of the joint analysis presented in Sec. IV A 2 are, thus, resolved, enabling the analysis of complex kinetic networks by smFRET. Practically, the analysis can be limited by the signal-to-noise ratio of the experimental data, which affects the quality of the resulting filters and, thus, the separation of the species, deteriorating the signal-to-noise ratio of the resulting correlation functions. Another practical challenge is the identification of the contributing species for the design of the filters. Here, FRET-lines are essential to identify and assign static and dynamic species from the two-dimensional histograms. To verify that the proper solution is attained, control simulations of ambiguous solutions could be performed, enabling a direct comparison of the experimental two-dimensional histograms to that of the obtained solutions.^33^

### C. Workflow for the analysis of multi-state kinetic networks

**FIG. 9.**
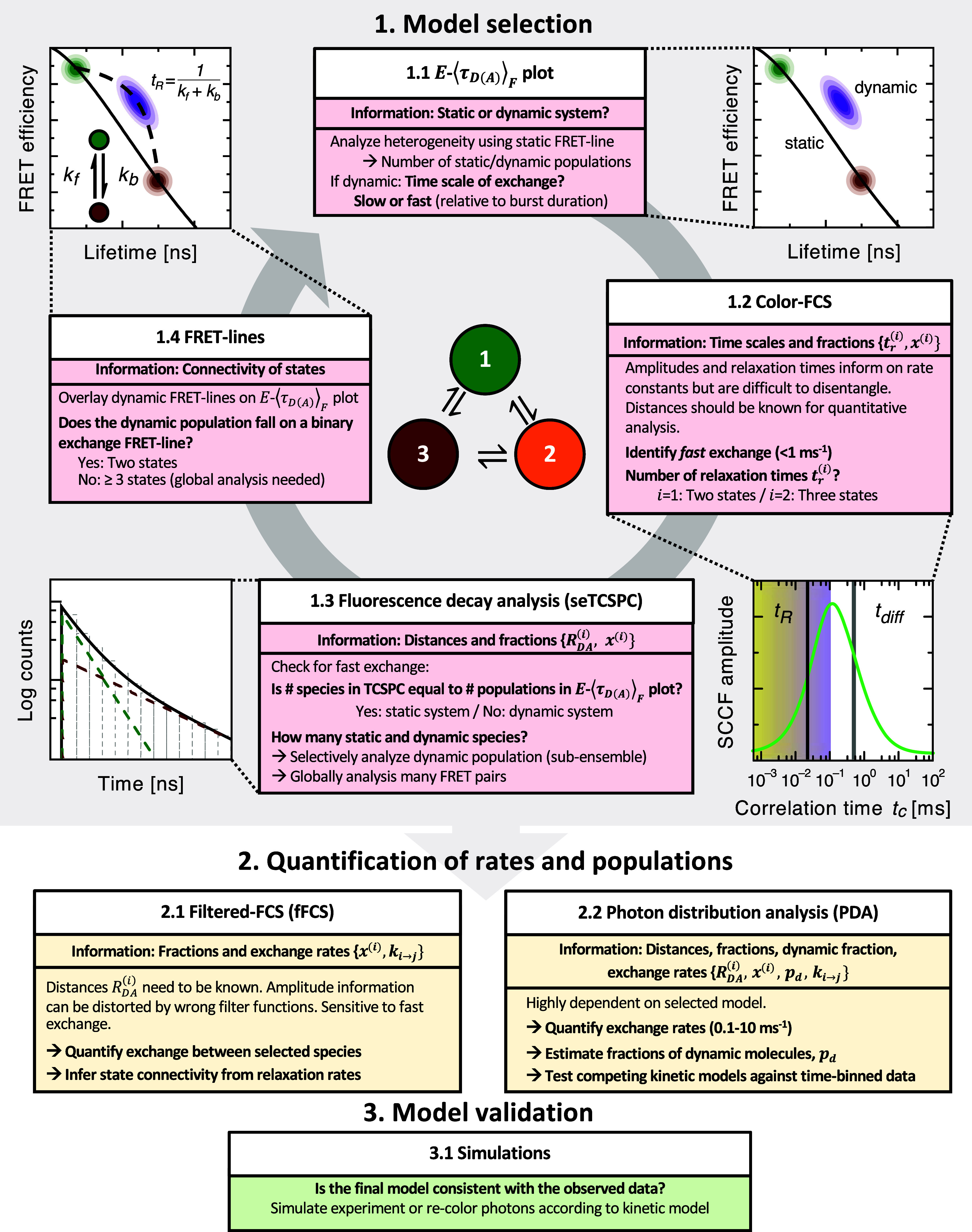
Workflow for the analysis of multi-state kinetic networks in time-resolved smFRET experiments. Step 1: Candidate models are derived from the combined information provided by the *E*–${\left\langle \tau_{D\left(A\right)}\right\rangle }_{F}$ plot, FRET-lines, seTCSPC, and color-FCS. Step 2: After the number of conformational states, their interdye distances and kinetic connectivity are defined, a model-based analysis by fFCS or PDA is applied to quantify the exchange rates and populations. Step 3: Finally, the model should be validated against the experimental data to resolve remaining ambiguities, e.g., using simulations of smFRET experiments. Schematics on spectroscopic data were adapted from Ref. 33.

To summarize the insights gathered here, we provide a general workflow for the analysis of multi-state dynamics in time-resolved single-molecule FRET experiments that encompasses three steps: (1) model selection, (2) quantification of exchange rates, and (3) model validation (Fig. 9).

The analysis starts with an inspection of the *E*–${\left\langle \tau_{D\left(A\right)}\right\rangle }_{F}$ plot (or equivalent transformations of the data, such as the moment representation introduced in Paper I of this Tutorial series^28^). In this first step, static and dynamic populations are identified based on their position with respect to the static FRET-line. The number and FRET efficiencies of static species can directly be estimated. For slow dynamics with respect to the timescale of diffusion, a trailing between dynamic and pseudo-static populations on the static FRET-line provides information on the involved limiting states. On the other hand, a defined averaged population that is shifted from the static FRET-line indicates fast conformational dynamics on the microsecond timescale.

Next, in step 1.2, a color-FCS analysis is performed to provide information on the timescales of the dynamic processes, allowing the detection or confirmation of fast dynamics on the microsecond or sub-microsecond time scale. The number of relaxation times additionally provides information on the number of kinetic states in the network, whereby a network with *N* states exhibits *N* − 1 relaxation times. Beyond this qualitative information, a quantitative analysis of the color-FCS curves requires precise knowledge of the FRET efficiencies of the species for a correct interpretation of the correlation amplitudes. For quantitative analysis, an fFCS analysis is often advantageous as described below.

**FIG. 10.**
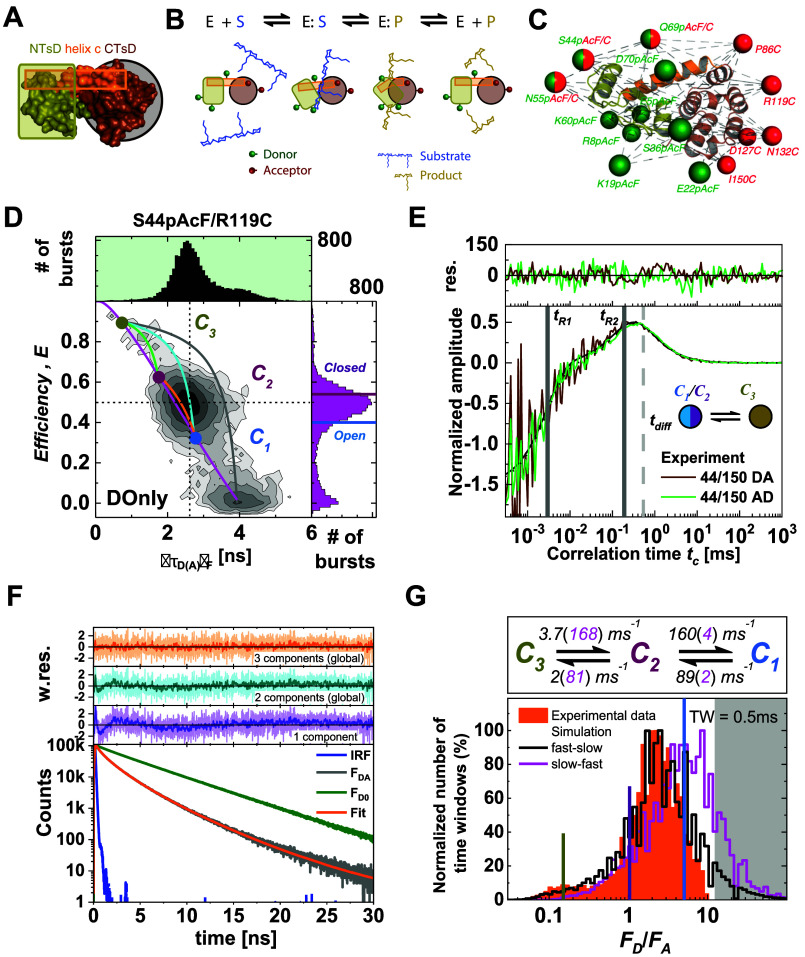
Integrative analysis of the conformational cycle of T4 lysozyme. (a) Subdomain architecture of Bacteriophage T4 lysozyme (T4L). Helix c connects the N-terminal and C-terminal subdomains, so that a variable reaction cavity is created via a hinge motion. (b) An extended Michaelis–Menten scheme is used to describe the enzymatic cycle of T4L. During the cycle of the substrate peptidoglycan (blue), the N-terminal (green) and the C-terminal subdomain (brown) come into close contact, resulting in a change of the FRET efficiency. (c) The 33 measured distinct FRET variants of T4L. The donor (D) Alexa488-hydroxylamine and acceptor (A) Alexa647-maleimide are coupled to *para*-acetylphenylalanine (pAcF) and cysteine (C), respectively. The spheres represent the average donor (green) and acceptor (red) position on the structure of T4L (PDB ID: 172L). (d) The *E*–${\left\langle \tau_{D\left(A\right)}\right\rangle }_{F}$ plot of the T4L variant labeled specifically at position S44 with the dye Alexa488 and at position R119 with the dye Alexa647 reveals the presence of three conformational states (C_1_, blue; C_2_, red; C_3_, green). The main population, indicated by the dashed lines, falls next to the static FRET-line (magenta), indicating fast exchange between the states C_1_ and C_2_. The state C_3_ at high FRET efficiency shows as a pseudo-static population that trails toward the main peak. Binary dynamic FRET-lines between the different states are shown in green (C_2_–C_3_), orange (C_1_–C_2_), and cyan (C_1_–C_3_). The interdye distances of the conformational states were obtained from a global TCSPC analysis of 33 FRET pairs [see panel (b)]. The gray line connecting to the donor-only (DOnly) population describes molecules with a high FRET efficiency for which the acceptor bleached during the observation time. The FRET efficiencies of the x-ray structures for the open (blue, PDB ID: 172L) and closed (violet, PDB ID: 148L) states are shown as horizontal lines in the FRET efficiency histograms. (e) Normalized species cross correlation functions for the T4L variant S44pAcF/I150C labeled with the donor at position 44 and the acceptor at position 150 (AD) or vice versa. The global fit with other variants shows two relaxation times (*t*_R1_ = 4.0 ± 2.3 *µ*s, *t*_R2_ = 230 ± 28 *µ*s) and a diffusion time *t*_diff_ = 0.54 ms. (f) The fluorescence decays of all 33 FRET variants were globally analyzed assuming one, two, or three conformational states. Shown is the experimental fluorescence decay of the variant S36pAcF/P86C (F_DA_, gray, overlaid in orange with best fit), the corresponding donor-only reference sample (F_D0_, green), and instrumental response function (IRF, blue). The weighted residuals (w.res.) for the one-state (violet), two-state (cyan), and three-state (orange) models are shown above. All 33 FRET pairs were globally analyzed with respect to the species fractions of the conformational states. (g) Comparison of the experimental histogram (orange) of the ratio of donor signal over acceptor signal, F_D_/F_A_, with the predicted histograms for the two competing linear three-state models with slow (magenta) or fast (black) exchange between the states C_1_ and C_2_. The exchange rates of the two models are given above. The gray shaded area indicates the region of donor-only events. Figures were adapted from Ref. 33.

Quantitative information on the interdye distances and species fractions of the different conformational states (Figs. 1) can be obtained from a TCSPC analysis in step 1.3. It is often challenging to infer the correct number of species from an analysis of the total fluorescence decay. Instead, it is advantageous to perform a species-selective sub-ensemble analysis (seTCSPC) of the different populations detected in the *E*–${\left\langle \tau_{D\left(A\right)}\right\rangle }_{F}$ plot. For dynamic populations, the number of decay components reports on the number of species involved in the dynamic exchange. In addition to an seTCSPC analysis, a global analysis of the fluorescence decays of many FRET pairs can reliably resolve the number of conformational states as shown for the example in Fig. 10.

In step 1.4, we use the information gathered on the number of conformational states, their interdye distances, and the relevant timescales for the dynamic exchange from the FCS and TCSPC analyses, to return to the *E*−${\left\langle \tau_{D\left(A\right)}\right\rangle }_{F}$ plot and distinguish between competing kinetic models based on the predicted dynamic FRET-lines (see Paper I of this Tutorial series^28^). Specifically, if a dynamic population does not fall on any of the potential binary dynamic FRET-lines, multi-state dynamics should be considered. This cycle should be repeated until one or more candidate models have been found that agree with the experimental data.

In step 2, a detailed quantitative analysis of the candidate models provides an estimate of the exchange rates. Based on the estimated FRET efficiencies of the involved conformational states, fFCS curves can be computed that provide information on the exchange rates and the connectivity of states. This information can be used to refine the candidate models by eliminating exchange pathways. For the remaining candidate models, ambiguities may be resolved by a PDA of the FRET efficiency histograms. PDA also provides further quantification of slow exchange rates (milliseconds and sub-millisecond time range) that are difficult to determine in the fFCS analysis. Finally, in step 3, the final model should be validated, e.g., by performing simulations^33^ or by recoloring of the detected photon events,^35^ to confirm that the observed data are compatible with the inferred kinetic model.

As an exemplary application to experimental data, we illustrate the discussed workflow using our recent integrative analysis of the conformational dynamics of the enzyme T4 lysozyme using 33 different FRET pairs^33^ [Figs. 10(a)–10(c)]. In view of the extended Michaelis–Menten scheme with all essential reaction states of an enzyme (*N* ≥ 3), we wanted to study the question for the example of T4 lysozyme (T4L) to which extent the minimal number of three reaction states correlates with number of resolvable conformational states.

Indeed, in addition to the well-known open and closed states of T4L (C_1_ and C_2_), we identified a third transiently populated intermediate conformational state C_3_ in the enzymatic cycle of T4L that has eluded previous crystallographic studies and was also detected in recent nuclear magnetic resonance (NMR) studies.^58^ Already in step 1.1 of our workflow, the existence of a third conformational state C_3_ is immediately evident from the *E*–${\left\langle \tau_{D\left(A\right)}\right\rangle }_{F}$ plot of the T4L mutant S44pAcF/R119C [Fig. 10(d)]. The two major conformational states C_1_ and C_2_ correspond to known crystal structures and are in fast exchange on the microsecond timescale, as evident from the displacement of the major population from the static FRET-line. In addition, the lowly populated state C_3_ is visible as a pseudo-static population on the static FRET-line at a FRET efficiency of ∼0.9, with a clear trailing toward the main population at *E* ∼ 0.5. In step 1.2, an fFCS analysis between the mixed C_1_/C_2_ population and the C_3_ state further revealed two relaxation times of *t*_*R*1_ = 4 *µ*s and *t*_*R*2_ = 230 *µ*s, supporting the existence of at least three conformational states [*N* ≥ 3, Fig. 10(e)]. In step 1.3, further evidence for the existence of a third conformational state was obtained in step 1.4 by a global analysis of the fluorescence decays (TCSPC) obtained for the 33 FRET pairs [Fig. 10(f)]. A two-component fit provided an inadequate description of the data and distances that were incompatible with the known structures of the open and closed states, whereas a three-component fit described all data well and yielded meaningful distances. In step 1.4, we revisit the *E*−${\left\langle \tau_{D\left(A\right)}\right\rangle }_{F}$ plot, draw FRET-lines that correspond to the suggested exchange network, and judge consistency with the experimental data. We recognize, that in view of the found fast relaxation times, it would not be expected to observe a pseudo-static population of the C_3_ state in the *E*−${\left\langle \tau_{D\left(A\right)}\right\rangle }_{F}$ plot. However, we clearly observed a population of the C_3_ state on the static FRET-line at *E* ∼ 0.9 that accounts for 5% of the detected burst [Fig. 10(d)]. Given that the equilibrium fraction of the C_3_ state was estimated at 20% from the global TCSPC analysis, we can conclude that a fraction of the T4L molecules must be in a pseudo-static state in the C_3_ conformation that does not participate in the dynamic exchange.

In step 2, the detailed analysis of the gathered kinetic and structural information suggested a three-state system that may be either cyclic (i.e., fully connected) or linear. There are two main arguments why a cyclic model can be discarded: (i) Considering all measured FRET variants, single-molecule events that fall on the respective C_1_ ⇋ C_3_ dynamic FRET-line were not observed; and (ii) using the structural information obtained by TCSPC, direct transitions between the structurally most compact C_3_ state to the most open C_1_ state (i.e., without going through the structurally intermediate state C_2_) are structurally and energetically infeasible. Hence, a linear three-state model with a gradual compaction that proceeds from the open C_1_ state through the intermediate C_2_ state to the most compact state C_3_ (C_1_ ⇋ C_2_ ⇋ C_3_) was found to be most likely. With the species fractions obtained from the TCSPC analysis and the relaxation times obtained by fFCS, however, two competing solutions were obtained, where the exchange between the states C_1_ and C_2_ could either be slow or fast. To solve this ambiguity, simulations of the two possible kinetic models were performed in the final step, step 3, to compare the predicted FRET efficiency histograms with the experimental results [Fig. 10(g)]. This clearly showed that the model for slow exchange between the states C_1_ and C_2_ disagrees with the experimental data.

**FIG. 11.**
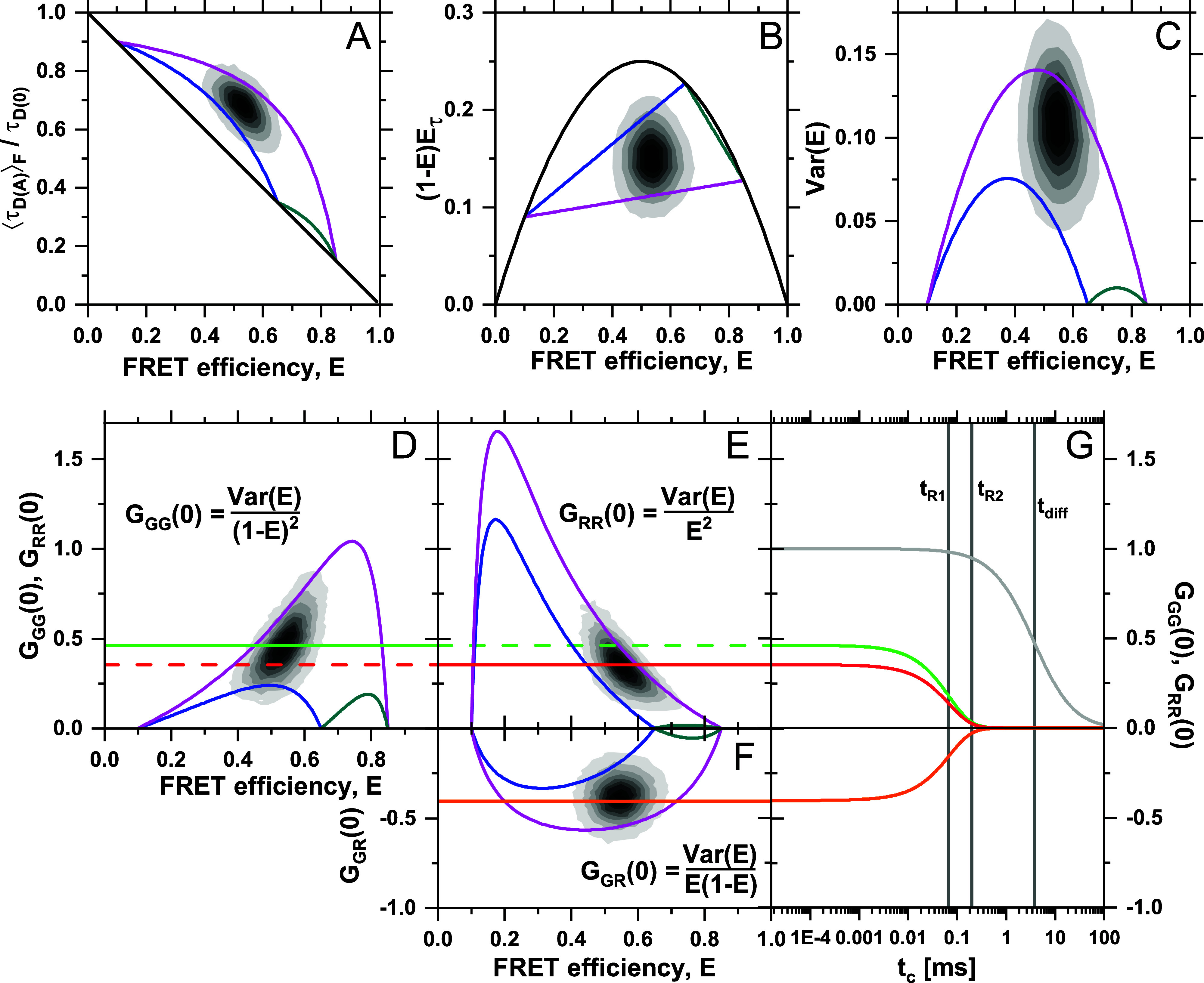
The relationship between correlation amplitudes and the FRET indicators *E* and ${\left\langle \tau_{D\left(A\right)}\right\rangle }_{F}$. A linear three-state system MF ⇌ LF ⇌ HF with exchange rates *k*_*i*→*j*_ = 5 ms^−1^ (simulation 7) and a diffusion time *t*_diff_ = 3.8 ms shows a single population that falls between the limiting dynamic FRET-lines in the ($E,{\left\langle \tau_{D\left(A\right)}\right\rangle }_{F}$) parameter space (a), the moment representation (b), and the variance representation (c). By normalizing the variance by the product of the average signals, the correlation amplitudes *G*_*GG*_(0), $G_{RR}\left(0\right)$, and *G*_*GR*_(0) can be estimated for each single-molecule event (d)–(f). The estimate of the amplitudes obtained from the single-molecule data agrees with the correlation amplitudes of the FCS curves (g). Static FRET-lines are given in black, and binary exchange lines in the various representation are colored blue for the exchange LF ⇌ MF, magenta for LF ⇌ HF, and turquoise for MF ⇌ HF. The kinetic contributions to the donor and acceptor auto- and cross correlation functions are given in green (G_GG_), red (G_RR_), and orange (G_GR_). The relaxation times of the FCS curves are *t*_*R*1_ = 67 *µ*s and *t*_*R*2_ = 200 *µ*s. In (g), the contribution of diffusion to the correlation functions is shown as a gray curve, and the kinetic relaxation times and timescale of diffusion are indicated as vertical gray lines.

In summary, we could trap T4L in distinct reaction states and determined the exchange rates and species fractions for each conformer. This correlation between the conformer fractions and distinct reaction states highlighted their functional relevance. Based on the observed connection between conformer populations and reaction states, we suggested that the conformer C_3_ is a new conformationally excited state of the enzyme with a compact structure that could be related to the product release species *E*:*P* [Fig. 10(b)]. This example for T4L shows that integrated quantitative single-molecule FRET-studies can be a valuable tool for dynamic structural biology^1^ by resolving the behaviors of long- and short-lived conformational states.

### D. Connecting FCS amplitudes and FRET indicators

We have shown how the combined information from FCS, TCSPC, and FRET-lines can resolve ambiguities in the analysis. The single-molecule information encoded in the two-dimensional histograms has been used to estimate the kinetic connectivity graphically, but its full potential has not been exploited. We derive the relationship between the two-dimensional histograms and the correlation amplitudes in color-FCS, which provides an additional restraint to the analysis.

The kinetic correlation functions represent the time-dependent (co)variance of the signals. The initial amplitude at zero lag time *t*_*C*_ may, thus, be expressed as

$$G_{ab}\left(t_{c}=0\right)=\frac{\left\langle S_{a}\left(t\right)S_{b}\left(t\right)\right\rangle }{\left\langle S_{a}\left(t\right)\right\rangle \left\langle S_{b}\left(t\right)\right\rangle }=\frac{Cov\left(S_{a},S_{b}\right)}{{\bar{S}}_{a}{\bar{S}}_{b}},$$ (33)

where Cov(*S*_*a*_, *S*_*b*_) is the covariance between the signals *S*_*a*_ and *S*_*b*_. In the ideal case, the signals in the donor and acceptor channel are defined by the FRET efficiency,

$$\begin{array}{c} S_{G}\propto 1-E,\\ S_{R}\propto E, \end{array}$$ (34)

resulting in the following expressions for the amplitudes of the correlation functions:

$$\begin{array}{c} G_{k,GG}\left(0\right)=\frac{\mathrm{Var}\left(E\right)}{{\left(1-E\right)}^{2}},\\ G_{k,RR}\left(0\right)=\frac{\mathrm{Var}\left(E\right)}{E^{2}},\\ G_{k,GR}\left(0\right)=-\frac{\mathrm{Var}\left(E\right)}{\left(1-E\right)E}, \end{array}$$ (35)

where we have used the relationship $\mathrm{Var}\left(E\right)=\mathrm{Var}\left(1-E\right)=-Cov\left(E,1-E\right)$. Thus, the correlation amplitudes represent the normalized variance of the FRET efficiency.

In the first part of the paper, we have shown that the variance of the lifetime or FRET efficiency distribution can be determined from the FRET observables *E* and ${\left\langle \tau_{D\left(A\right)}\right\rangle }_{F}$ by

$$\mathrm{Var}\left(E\right)=\left(1-E\right)\left(E-E_{\tau }\right),$$ (36)

where $E_{\tau }=1-\frac{{\left\langle \tau_{D\left(A\right)}\right\rangle }_{F}}{\tau_{D\left(0\right)}}$ is the FRET efficiency calculated from the intensity-weighted average lifetime. This implies that we can calculate the amplitudes of the correlation function directly from the single-molecule FRET indicators, under the condition that the dynamics are fast compared to the diffusion time. For slower dynamics, the variance is underestimated due to the limited sampling within a single-molecule event (see Sec. III).

The connection between single-molecule FRET indicators and the correlation amplitudes is illustrated in Fig. 11 for a three-state system with fast dynamics in the absence of static species, showing a single population that falls between the binary FRET-lines in the ($E,{\left\langle \tau_{D\left(A\right)}\right\rangle }_{F}$), moment, or variance representations [Figs. 11(a)–11(c)]. Using Eq. (35), a molecule-wise estimate of the correlation amplitudes is obtained that can be compared to the actual correlation amplitudes obtained from FCS analysis [Figs. 11(d)–11(g)]. The static and dynamic FRET-lines can likewise be converted into the equivalent of FCS amplitudes [Figs. 11(d)–11(f)]. The information encoded in the single-molecule FRET indicators could be used as an additional restraint in the analysis.

## V. CONCLUSIONS

Using synthetic and experimental datasets, we challenged the capabilities of conventional analysis methods used in smFRET experiments that rely on one-dimensional data representation. In particular, the coexistence of static and dynamic FRET species (see Fig. 1) complicated the kinetic analysis (Fig. 1). In our integrative approach, FRET-lines serve as visual guidelines for interpreting experiments and for classifying populations of single molecules as static or dynamic. For slow exchange kinetics, FRET-lines directly resolve the connectivity of states. For kinetics that is significantly faster than the integration time, molecule-wise histograms, combined with FRET-lines, help distinguish dynamic averages from static populations. We developed a global analysis framework of FCS and TCSPC, which was not sufficient to identify unique solutions for two-state kinetic networks in the presence of static states. While it was possible to detect the presence of dynamics and quantify their timescale, the network connectivity and the corresponding static and dynamic fractions were not unambiguously recovered. Here, FRET-lines provided the required information to identify the limiting states of the dynamic exchange and their connectivity within the kinetic network. As a next step, the global analysis framework could be extended to utilize species-correlation function from filtered-FCS and include photon distribution analysis. We also showed that the equilibrium constant of dynamic processes could be estimated from a graphical analysis of the *E*−${\left\langle \tau_{D\left(A\right)}\right\rangle }_{F}$ plot even in the presence of a background of static molecules. Together with the relaxation time obtained by FCS, the microscopic rate constants can, thus, be quantified without requiring a precise determination of the FCS amplitudes. More complex kinetic networks consisting of three fast-exchanging states could be resolved by fFCS, providing species-specific auto- and cross correlation curves that reveal the connectivity from the patterns of the relaxation times and amplitudes. Unlike color-FCS, the number of species-correlation functions in fFCS increases with the number of states, allowing robust analyses of multi-state networks.

Our global analysis framework is a step toward a self-consistent, holistic model of the two-dimensional histogram of the observables *E* and ${\left\langle \tau_{D\left(A\right)}\right\rangle }_{F}$ in smFRET experiments. The prediction of the molecule-wise distribution of these parameters currently relies on Monte Carlo simulations, which introduce stochasticity into the analysis that poses a problem for most optimization algorithms. Although specialized algorithms from the field of machine learning, such as evolutionary algorithms or simulated annealing, may be used to overcome the convergence problem, these algorithms require many iterations for convergence. Deterministic and efficient algorithms are therefore needed. We envision that the future of smFRET studies will rely on a holistic analysis of the complete experimental information, wherein the kinetic information encoded in the multidimensional histograms of molecule-wise parameters will be an essential first step for proposing candidate models that are subjected to further analysis. Using this approach, it will be possible to quantify the kinetics in complex networks, paving the way toward understanding the intrinsic dynamics of biomolecules and addressing fundamental questions relating to their function.

## SUPPLEMENTARY MATERIAL

See the supplementary material for additional information on potential ambiguities in multi-state systems, detailed derivations of the dependence of the dynamic shift on the species fractions and the state occupancy distribution as discussed in Sec. III, the full derivation of the FCS model functions used in this work, additional discussion of the observed ambiguities in the global FCS and TCSPC analysis, a description of the simulation algorithm and parameters, the analysis of simulation 1 comprised of four static states, details on the photon distribution analysis of simulation 2, and the species autocorrelation functions of simulations 4–7.

## Data Availability

The simulated data that support the findings of this study are available from the corresponding authors upon reasonable request. The simulation settings are described in detail in Sec. 7 of the supplementary material. To support the wide use of FRET-lines presented in this Tutorial, we provide extensive software to generate the lines for different user levels. Computational tools for the calculation of FRET-lines discussed in this work in the Python programming language are available at https://github.com/Fluorescence-Tools/FRETlines, Ref. 59. The repository includes example Jupyter notebooks as direct tutorials on how to generate the different FRET-lines step-by-step by an interactive exploration of static, dynamic, and the different polymer FRET-lines. As a second tool, we provide a graphical user interface in the program “FRET-lines explorer” that is available with the software package for multiparameter fluorescence spectroscopy available at https://www.mpc.hhu.de/software/mfd-fcs-and-mfis, Ref. 60, and separately as the supplementary material of Ref. 28.

## NOMENCLATURE

**Used symbols and definitions. Definitions in smFRET experiments of multi-state systems**
*C*^(1*s*)^, *C*^(2*s*)^: pseudo-static conformational (structural) state of the biomolecule
*C*^(1*d*)^, *C*^(2*d*)^: dynamic conformational (structural) state of the biomolecule
*O*^(1)^, *O*^(2)^: observed fluorescence species defined by a unique set of fluorescence properties
*P* ^(1,2)^: population in the experiment originating from dynamic mixing of species 1 and 2

**Graphical analysis of kinetics**
ds: dynamic shift of a population orthogonal to the static FRET-line
$E^{\left(i\right)}$: FRET efficiency of species *i*
⟨*E*⟩_exp_: average FRET efficiency over all single-molecule events of the experiment
*E*_*m*_: modal value of the FRET efficiency distribution
$I_{0}\left(x\right),I_{1}\left(x\right)$: modified Bessel functions of the first kind of order zero and one
*K*: equilibrium constant of dynamics
*k*: sum of the microscopic rate constants *k* = *k*_12_ + *k*_21_
*k*_12_, *k*_21_: exchange rates between states 1 and 2
*P*(*E*), $P\left(x^{\left(1\right)}\right)$: probability to observe a given average FRET efficiency or state occupancy of state 1 within a single-molecule event
*T*: observation time/integration time
*x* ^(*i*)^: state occupancy (fraction) of species *i*
$x_{d}^{\left(i\right)}$: equilibrium species fraction of species *i* in a dynamic system
*x*_*m*_: modal value of the state occupancy distribution
$x_{d,\lim}^{\left(i\right)}$: limiting equilibrium fraction at *x*_*m*_ = 0
$\xi_{12}\left(x^{\left(1\right)}\right)$: dynamic part of the state occupancy distribution

**Description of correlation functions**
$A_{ab}^{\left(l\right)}$: *l*th pre-exponential factor of the kinetic correlation function between signals *a* and *b*
Cov(*x*, *y*): covariance of the quantities *x* and *y*
$\bar{E}$: species-averaged FRET efficiency
$G_{ab}\left(t_{c}\right)$: correlation function of channels *a* and *b*
$G_{\text{diff}}\left(t_{c}\right)$: diffusion correlation function
$G_{k,ab}\left(t_{c}\right)$: kinetic correlation function between the channels *a* and *b*
**K**: transition rate matrix
LF, MF, HF: low-FRET, medium-FRET, and high-FRET species
*N*: average number of particles in the confocal volume
*p*_*d*_: global fraction of dynamic molecules
*Q*_0_: molecular brightness of the donor in the absence of FRET
***q***_*G*_, ***q***_*R*_: apparent brightness in the green (G) and red (R) detection channels
$S_{a}\left(t\right)$: signal in channel *a* at time *t*
***S***_*a*_: vector of the signals of the different species in channel *a*
$\bar{S_{a}}$: species-averaged signal in channel *a*
*t*_*c*_: correlation lag time
$t_{R}^{\left(l\right)}$: *l*th relaxation time (inverse of the negated *l*th eigenvalue of the transition rate matrix)
*t*_diff_: diffusion time
$\mathrm{Var}\left(x\right)$: variance of the quantity *x*
*w*_*TCSPC*_, *w*_*FCS*_: weights used in the analysis of TCPSC decays and FCS curves
*w*_0_, *z*_0_: lateral and axial width of the confocal volume
***x***: vector of the total species fractions *x*^(*i*)^
***x***_*d*_, ***x***_*s*_: vector of the dynamic and pseudo-static species fractions
**X**_*d*_, **X**_*s*_: diagonal matrices of the dynamic and static species fractions
**Γ** ^(*l*)^: *l*th eigen-matrix of the transition rate matrix
λ^(*l*)^: *l*th eigenvalue of the transition rate matrix
$\chi_{\text{globa}l}^{2}$, $\chi_{\mathrm{TCSPC}}^{2}$, $\chi_{\mathrm{FCS}}^{2}$: reduced chi-squared values of the global analysis
$\partial_{ab}^{\left(ij\right)}$: normalized contrast factor between species *i* and *j* of the correlation function between signals *a* and *b*
⟨⋯⟩: time average over a long measurement
